# Prospect of extracellular vesicles in tumor immunotherapy

**DOI:** 10.3389/fimmu.2025.1525052

**Published:** 2025-02-26

**Authors:** Wenbo Xia, Yunhan Tan, Yongen Liu, Na Xie, Huili Zhu

**Affiliations:** ^1^ Department of Reproductive Medicine, Key Laboratory of Birth Defects and Related Diseases of Women and Children of Ministry of Education, West China Second University Hospital of Sichuan University, Chengdu, China; ^2^ State Key Laboratory of Oral Diseases and National Clinical Research Center for Oral Diseases, West China Hospital of Stomatology, Sichuan University, Chengdu, Sichuan, China; ^3^ West China School of Basic Medical Sciences and Forensic Medicine, Sichuan University, and Collaborative Innovation Center for Biotherapy, Chengdu, China

**Keywords:** extracellular vesicles, nanotechnology, tumor immunotherapy, drug delivery and targeting, immunotherapy combined therapy

## Abstract

Extracellular vesicles (EVs), as cell-derived small vesicles, facilitate intercellular communication within the tumor microenvironment (TME) by transporting biomolecules. EVs from different sources have varied contents, demonstrating differentiated functions that can either promote or inhibit cancer progression. Thus, regulating the formation, secretion, and intake of EVs becomes a new strategy for cancer intervention. Advancements in EV isolation techniques have spurred interest in EV-based therapies, particularly for tumor immunotherapy. This review explores the multifaceted functions of EVs from various sources in tumor immunotherapy, highlighting their potential in cancer vaccines and adoptive cell therapy. Furthermore, we explore the potential of EVs as nanoparticle delivery systems in tumor immunotherapy. Finally, we discuss the current state of EVs in clinical settings and future directions, aiming to provide crucial information to advance the development and clinical application of EVs for cancer treatment.

## 1 Introduction

Traditional cancer therapies, such as chemotherapy, radiotherapy, and surgery, aim to eliminate or directly remove cancer cells. However, these treatments often come with multiple adverse effects (1). Chemotherapy and radiotherapy, for instance, can impact normal cells, leading to immunocompromising effects and side effects like alopecia, nausea, and cytopenia. Surgery, while effective, may inadvertently damage normal tissue, potentially causing long-term complications. Moreover, surgical interventions have limited efficacy in treating metastatic cancer and may even accelerate the recurrence of tumors (2). In contrast, immunotherapies, including immune checkpoint inhibitor (ICI) treatments, adoptive cell immunotherapy (ACT), and tumor vaccines, have emerged as promising alternatives. These approaches aim to enhance antitumor immune responses, leveraging the host’s innate defense mechanisms to specifically target and eliminate malignant cells while minimizing off-target effects. Several ICIs, such as the CTLA-4 monoclonal antibody ipilimumab, programmed death-1 (PD-1) monoclonal antibodies nivolumab and pembrolizumab, and PD-L1 monoclonal antibodies atezolizumab and avelumab, have been approved for clinical use (3–7). However, ICIs may impair normal tissues such as gastrointestinal tract, thyroid, and lung (8, 9). Adoptive cell immunotherapy involves therapies like tumor-infiltrating lymphocytes (TILs), chimeric antigen receptor T cells (CAR-T), and TCR-modified T cells (TCR-T). While ACT can outperform traditional therapies in certain cases, its widespread use is hindered by complex and costly production processes (10). Tumor vaccines utilize tumor-specific antigens (TSAs) or neoantigens to induce acquired immunity against tumors. While their long-lasting antitumor effects make them suitable for patients with smaller tumors, the time-consuming production of tumor vaccines may pose challenges in keeping up with the progression of tumors (11). Despite these advancements, addressing the immune evasion tactics employed by cancer cells continues to be a significant challenge in the realm of tumor immunotherapy (12).

Given the intricate mechanisms by which cancers evade the immune system, employing combination therapies that address different phases of the cancer-immunity cycle may yield more successful outcomes. Recently, innovative drug delivery systems utilizing nanoparticles (NPs) and extracellular vesicles (EVs) have surfaced as comprehensive platforms for the concurrent delivery of multiple therapeutics. These systems aim to counteract immunosuppression and foster a tumor microenvironment (TME) that is supportive of immune responses (13). Various nanomaterials, including liposomes, nanostructured lipid carrier systems (NLCs), solid lipid nanoparticles (SLNs), hydrogels, nanoemulsions, polymer micelles, and inorganic NPs, have demonstrated potential as nanoplatforms for drug delivery. These materials offer significant advantages such as high bioavailability, controllable drug release, and remarkable kinetic stability (14, 15). EVs exhibit superior biocompatibility, transferability, and targeting ability compared to synthetic NPs (16). As small vesicles released by cells, they can be found in various bodily fluids, including blood, saliva, urine, cerebrospinal fluid (CSF), pleural fluid, and breast milk (17, 18). They possess intrinsic capabilities to penetrate barriers and induce functional alterations in targeted cells (19, 20). Notably, EVs can cross the blood–brain barrier (BBB), overcoming limitations for small-molecule drug passage (21). At the cellular level, EVs efficiently engage with the plasma membrane through various ligand/receptor interactions, leading to enhanced internalization compared to synthetic nanocarriers (22–24). Internalization primarily occurs through endocytosis, with distinct pathways identified for different cell types. This efficient cargo delivery to recipient cells suggests a promising role for EVs in delivering antigens or drugs for cancer therapy (25, 26).

Beyond drug delivery, EVs play an essential role in tumor therapy by virtue of their high immunostimulatory factors, regulating inflammatory reactions and adjusting immune function (27). Bioactive molecules within EVs, including proteins, RNAs, DNAs, lipids, amino acids, and metabolites, modulate intercellular communication and influence the TME (28). Tumor EVs, dendritic cells (DCs), and antigen-presenting cell (APC)-derived EVs work together, consisting of a vaccination platform supporting DC maturation and antigen presentation. EVs imitate the function of their donor cells, making them a potential alternative for adoptive cell therapy (ACT). Engineered EV surfaces make the delivery more targeted, since EVs can cross the BBB and blood–tumor barrier (BTB). Moreover, the artificially loaded cargoes further promoted the intrinsic antitumor capacity of EVs (29). However, despite advantages in the field of EVs, there are still some problems that need to be solved in the future, which may hinder the application and effectiveness of EVs in cancer treatment.

A comprehensive understanding of EVs and their interactions with cells is crucial for their application in anticancer treatment. We begin with a brief overview of the biogenesis and current modification strategies of EVs, emphasizing their functions in the TME. The subsequent section reviews the current application strategies of EVs in cancer therapy. Additionally, we discuss the challenges and potential solutions regarding the clinical use of EVs. Ultimately, our aim is to provide essential information to promote the development and clinical application of EVs in cancer treatment.

## 2 Biogenesis and modification of EVs

### 2.1 Biogenesis of EVs

**Figure 1:**
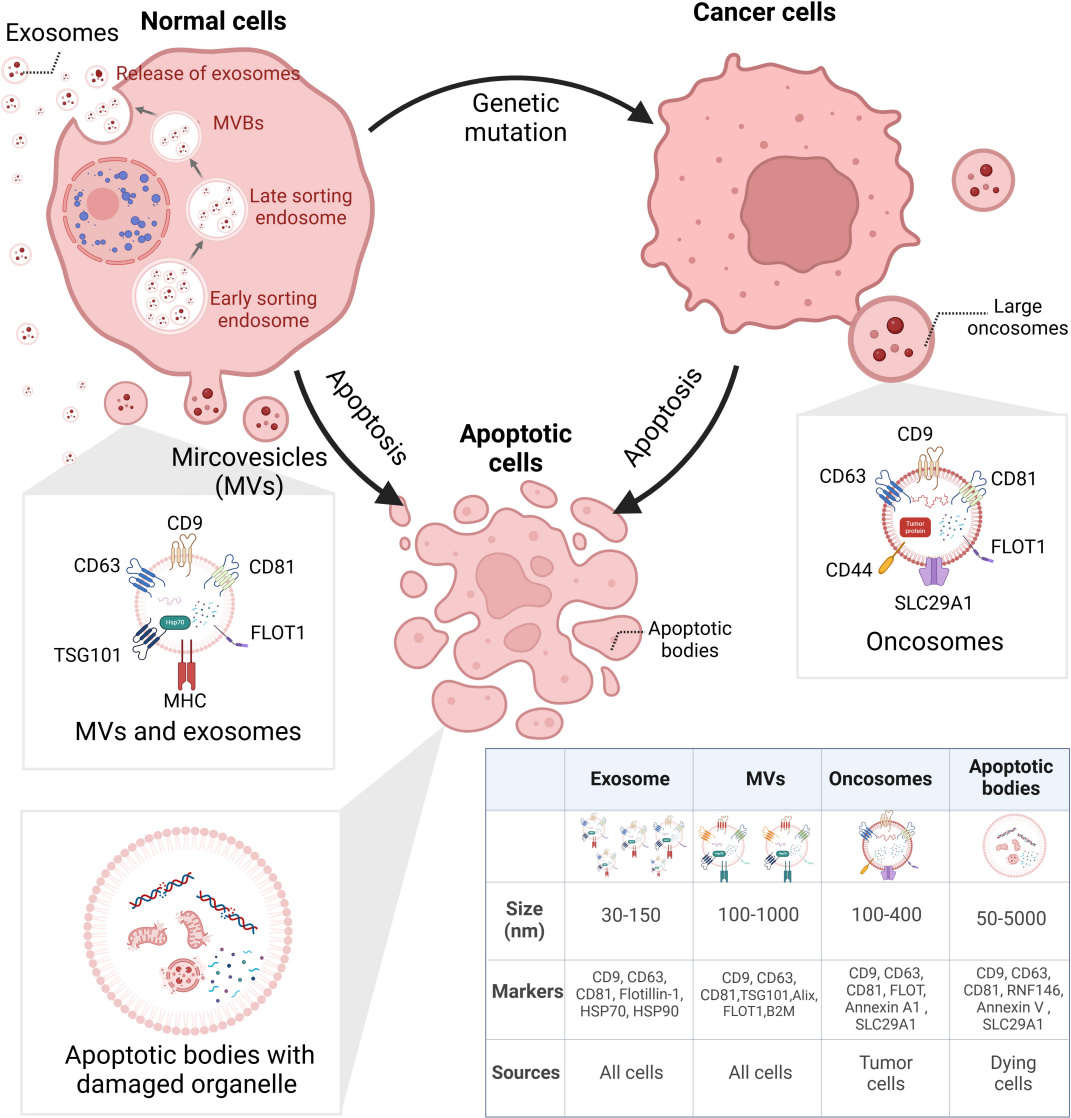
Biogenesis of heterogeneous EVs. EVs can be categorized via their biogenesis, which distinguishes their size, surface markers, and cargoes. EXOs derived from small bulbs emerged in early sorting endosomes. After selection in the last sorting endosomes, EXOs are released from MVBs. Different from EXOs, MVs are directly released from cells. LOs are large vesicles secreted by cancer cells, containing various tumor-specific factors. ABs are bubbles separated from dying cells; inside are broken organelles and apoptosis-related components. EVs, extracellular vesicles; EXOs, exosomes; MVBs, multivesicular bodies; MVs, microvesicles; LOs, large oncosomes; ABs, apoptosis bodies. BioRender was used to create the figure.

EVs are NPs derived from different cell activities with heterogeneity. According to Minimal Information for Studies of Extracellular Vesicles (MISEV) 2023, the EVs generated from multivesicular bodies (MVBs) are classified as exosomes and those derived from cell membranes are named ectosomes, e.g., microvesicles (MVs) and microparticles. Some EVs are related to a specific type of cellular process including apoptotic bodies from programmed cell death, migrasomes from cell migration, and oncosomes from tumor progression (30). **Figure 1** illustrates the biogenesis of different types of EVs.

Furthermore, drugs and genetic intervention may contribute to the stimulation or suppression of EV release (31, 32). However, most isolation techniques are unable to enrich EVs of different biogenesis. The lack of universal biomarkers discourages definitive characterization of biogenesis-based subtypes. Therefore, the biogenesis-related terms such as exosomes and ectosomes are not encouraged to be applied unless they are specifically and carefully separated. However, the majority of the existing literature on “exosomes” and “ectosomes/microvesicles” refers to a broad population of EVs, rather than EVs originating from specific biogenesis pathways (30). In the article, the terms “EVs”, “exosomal”, and “exosome (EXO)” all refer to “extracellular vesicles” referred in MISEV2023 with no indication of their biogenesis.

### 2.2 Modification of EVs

**Figure 2:**
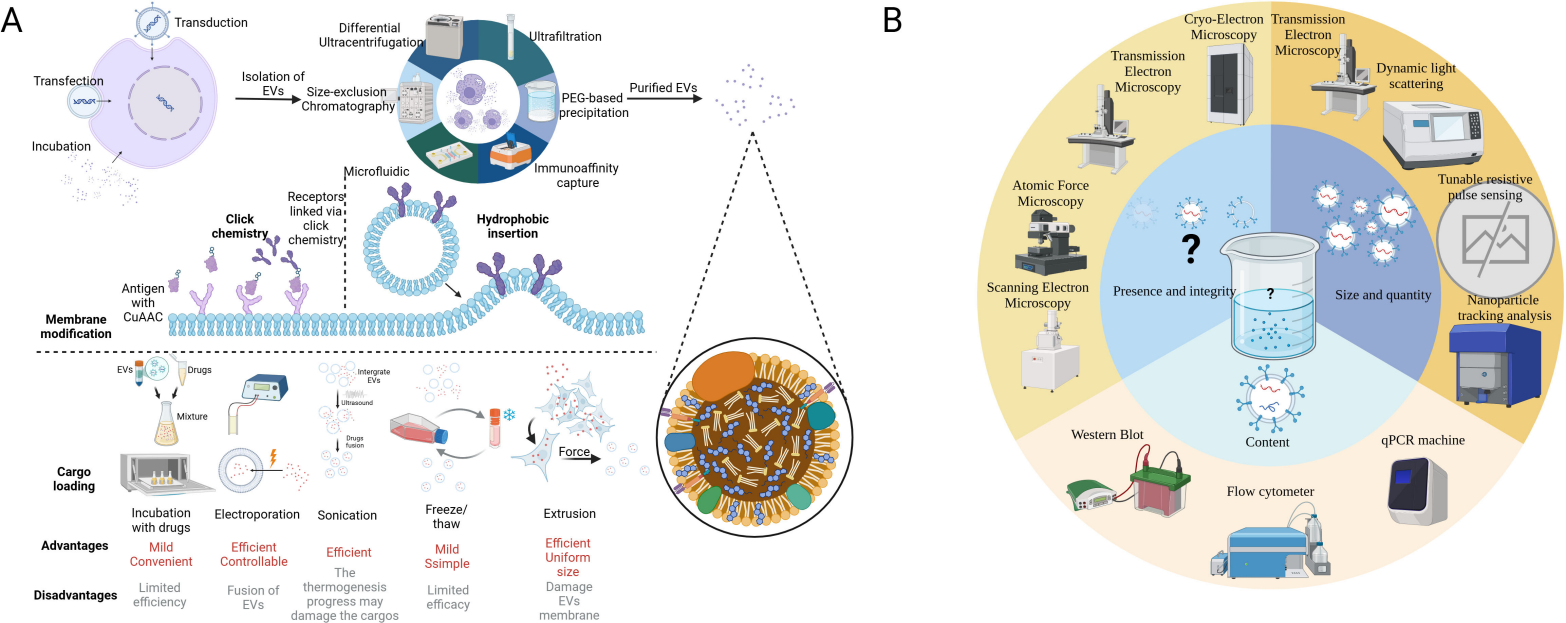
Modification technique of EVs and recommended procedure analyzing EV samples. **(A)** The figure illustrates techniques for processing EVs from extraction to modification and cargo loading. The first step is to extract EVs from the complex fluid (tissue fluid or culture medium), while dUC is the most common method. The surface membrane modification can be achieved by click chemistry and hydrophobic insertion, which enhances its targeting capacity. Finally, versatile techniques have been applied in the cargo loading of EVs according to the character of the cargo. In some cases, the donor cells are loaded with mRNAs or cocultured with drugs initially to generate EVs with special features. **(B)** According to MISEV2018, at least two methods are required to characterize the EVs. Microscopies can be applied in visualizing EVs. TEM, DLS, TRPS, and NTA measure the size and quantity of various EVs. WB, qPCR, and flow chemistry can analyze protein and nucleic acid in EVs. It is noted that there is no existing equipment that can easily characterize all the EVs in a sample (16). EVs, extracellular vesicles; dUC, differential ultracentrifugation; MISE2018, minimal information for studies of extracellular vesicles 2018; TEM, transmission electron microscope; DLS, dynamic light scattering; TRPS, tunable resistive pulse sensing; NTA, nanoparticle tracking analysis; WB, Western blot; qPCR, quantity polymerase chain reaction. BioRender was used to create the figure.

Apart from the naïve EVs that possess an intrinsic ability to target different cell types, such as tumor cells, immune cells, and stem cells, a number of synthetically modified EVs have been developed to improve their biodistribution and targeting capabilities, boosting EV-based tumor immunotherapy (19, 33). Surface modifications through cellular machinery techniques facilitate tumor targeting and intercellular transformation (34). Cargo-loaded EVs are now a new trend in tumor immunotherapy, which can prevent the clearance of drugs in blood, limit the dose, and reduce side effects (35, 36). Pre-loading is performed before EV secretion or isolation. It enables the membrane integrity of EVs and allows for continuous and easy production of EVs since donor cells are preserved (37). Post-loading directly loads cargo into EVs, exhibiting a higher loading efficiency but potentially altering membrane integrity (38). **Table 1** lists the common techniques for EV modification. **Figure 2** illustrates the modification and isolation techniques of EVs.

**Table 1:** Biogenesis and modification of EVs.

	Principle	Advantages	Disadvantages	Application	Reference
Membrane modification					
Genetic engineer	1. Design target molecules 2. Insert the corresponding gene to donor cells (transduction/transformation) 3. Extraction of EVs with specific molecules on membrane	1. Capacity to add complex and fragile ligands on surface 2. Genes can be easily designed	1. Some donor cells like red blood cells/stem cells can hardly to be transduced 2. Change of genes may induce unexcepted errors	1. Add fragile ligands 2. Add ligands from a selected gene	(350–352)
Chemical modification	1. Click chemistry combining target molecules with target cells with EDC/NHS coupling 2. Metabolic labeling involves incorporating reactive groups into EV membrane proteins or glycoproteins by culturing donor cells in a medium containing azide-bearing amino acids or azide-modified saccharides. This approach enables subsequent modifications with targeting moieties via click chemistry 3. Affinity binding applies affinity molecules on EV surface to link target moieties	1. Click chemistry is a robust binding technique 2. Metabolic labeling is stable and efficient 3. Affinity binding is easy to operate. Avoiding perturbation on EV surface	1. Click chemistry: The non-specific reaction may alter the properties of EVs 2. Metabolic labeling: Application of azide-bearing supplements for large-scale media and substrate synthesis in click chemistry is expensive 3. Affinity binding: Less robust compared with the other two techniques	1. Insertion of peptides, proteins, aptamers, and lipids	(353–356)
Cargo-loading techniques					
Pre-loading					
Co-incubation	1. Mix the donor cells with drugs 2. Yield EVs containing drugs	1. Easy to operate 2. Avoiding damages on EV surface	1. Limited efficiency, especially to hydrophilic drugs	1. Lipophilic drugs like doxorubicin (DOX) and paclitaxel (PTX) 2. Small molecules	(357–359)
Transfection	1. Transfection of the donor cells regulates expression of a given gene, inducing an alteration in EV content	1. Convenience in loading nucleic acids 2. Stability	1. Vector may get into the EVs, causing unwanted results 2. Alterations in gene expression and the toxicity of transfection agents can lead to changes in parental cells	1. Nucleic acids 2. Proteins and peptides	(360–362)
Regulation of microenvironment	1. Alteration of microenvironment (e.g., drug stimulation, changes in temperature, and oxygen concentration) induces secretion of EVs with different content	1. Easy to operate	1. Risk of cell death	1. Drug induction 2. Stress induction	(363, 364)
Post-loading					
Co-incubation	1. Directly co-incubate drugs with EVs	1. Easy to operate	1. Low efficiency 2. Limited scope of cargoes	1. Lipophilic drugs 2. Hydrophilic drugs encapsuled with lipid coat 3. Proteins and peptides	(365)
Sonication	1. Sonication deforms the vesicle membrane, facilitating cargo penetration	1. Efficient and simple	1. Damage to membrane integrity 2. Risk of vesicle aggregation 3. Degradation of nuclear acid	1. Drugs 2. Proteins 3. Nanomaterials	(366, 367)
Extrusion	1. In the technique, EVs and cargoes are mixed and compressed via a lipid extruder with 100- to 140-nm pores. During this process, the EVs membrane is disrupted, allowing cargoes to be encapsulated as the membrane reassembles	1. Efficient 2. Identical EV size after extrusion	1. Greatly alter membrane structure 2. Altered zeta potential and are cytotoxic	1. Drugs 2. Proteins 3. Nanomaterials 4. Nucleic acid	(368, 369)
Electroporation	1. Electroporation applies an electrical pulse to form pores in the EV bilayer membrane, allowing loading molecules to enter the vesicles	1. Optimized processes ensure high loading efficiency	1. Risk of vesicles/cargoes aggregation	1. Drugs 2. Proteins 3. Nanomaterials 4. Nucleic acid	(19, 370, 371)
Freeze/thaw	1. Repeated freeze–thaw cycles damage the vesicle membrane, enabling cargo diffusion	1. Simple procedures	1. Low efficiency 2. Risk of vesicle aggregation and vesicle protein damage	1. Proteins and peptides	(372)
Purification techniques					
Differential ultracentrifugation (dUC)	1. Utilizing a succession of centrifugal forces and durations to sequentially separate particles through sedimentation based on their size	1. Applicability for isolating EVs in large volume of biological liquids 2. Limited impact on EVs as no chemicals are used for EV isolation 3. High purity 4. Ease of operation 5. Good reproducibility	1. Requirement of expensive equipment 2. Presence of contaminants (partials of similar size) 3. Possible structure damage 4. Time-consuming	1. Large volume isolation 2. Purification of small EVs	(245, 373, 374)
Ultrafiltration (UF)	1. Isolating particles within a predetermined size range using membranes with defined pore sizes	1. Relatively less time 2. Absence of expensive equipment	1. Lower purity and yield compared with dUC 2. Poor RNA and mRNA preservation	1. EVs concentration 2. Size-oriented EVs separation	(373, 375–377)
Polyethylene glycol (PEG)-based precipitation	1. Wrapping EVs in an aqueous PEG solution to help exosome aggregates develop that enable them to be precipitated using low-speed centrifugation at 1,500 *g*	1. Production of pure exosomal fraction based on immunological markers 2. Application in clinical research settings	1. Contamination of co-aggregated substance	1. Large volume isolation	(373, 378)
Immunoaffinity capture	1. A technique separating EVs with specific surface proteins, especially tetraspanins like CD9, CD63, and CD81	1. High specificity and purity	1. High-cost antibodies 2. Elution could harm the natural EV structure. 3. Specificity limits its clinical use	1. Isolation of EVs with specific proteins on surface	(379–382)
Microfluidic	1. A high-throughput technique that use microfluidic tools to separate EVs including a number of criteria, such as immunoaffinity, size, and density	1. Fast processing speed 2. High level of purity	1.Complex and costly equipment 2. Shared disadvantages in immunoaffinity capture section	1. Integration of purification and examination	(383–385)
Size-exclusion chromatography (SEC)	1. Starting biofluid is applied as the mobile phase in this method, and a porous gel filtration polymer is used as the stationary phase. Because of the characteristics of the stationary phase, differential elution is possible: larger particles elute first, followed by smaller vesicles, followed by proteins that are not membrane-bound.	1. Better purity 2. Processing samples efficiently 3. Keep the integrity of EVs	1. Unable to differentiate between contaminates of the same size 2. Low yield	1. Isolation of fragile EVs	(305, 386–388)

## 3 The interplay of various cell-derived EVs in the TME

**Figure 3:**
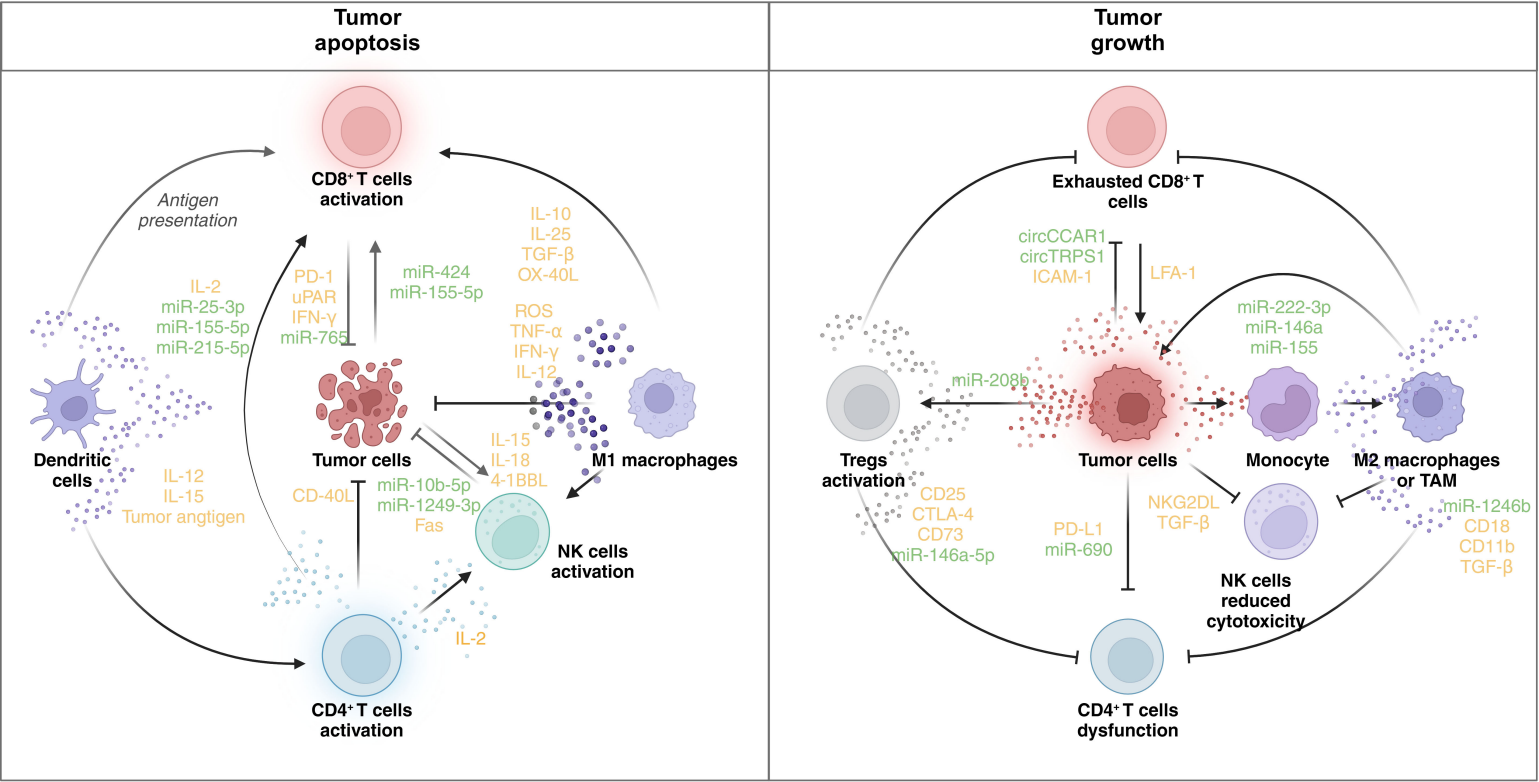
Immune regulatory role of EVs in the TME. The figure shows the regulatory network of EVs in the TME. In the left panel, the activation of CD8^+^ T cells, DCs, CD4^+^ T cells, NK cells, and M1 macrophages facilitates tumor cell death. DCs present tumor antigens and secrete IL-12 and IL-15, promoting the activation of CD8^+^ and CD4^+^ T cells. CD8^+^ T cells, when activated, release IFN-γ and other cytotoxic molecules, contributing to tumor apoptosis. CD4^+^ T cells secrete IL-2, further supporting CD8^+^ T-cell and NK cell activation. NK cells produce IL-15, IL-18, and 4-1BBL, enhancing their cytotoxic function. M1 macrophages release ROS, TNF-α, and IFN-γ, promoting an antitumor response. Tumor cells express molecules like CD40L and Fas, which enhance immune cell-mediated killing. Various microRNAs (e.g., miR-25-3p, miR-155-5p, and miR-1249-3p) are involved in modulating the immune response. In contrast, immune dysfunction promotes tumor progression. In the right panel, CD8^+^ T cells become exhausted, expressing inhibitory receptors (e.g., PD-1 and uPAR), and are unable to mount an effective antitumor response. Tregs are activated, suppressing immune activity through the expression of CD25, CTLA-4, and other immunosuppressive molecules. NK cells exhibit reduced cytotoxicity due to the influence of TGF-β and NKG2DL. Monocytes differentiate into M2 macrophages or TAMs, which secrete immunosuppressive cytokines (e.g., TGF-β) and promote tumor growth. Tumor cells themselves express PD-L1, further inhibiting immune responses. MicroRNAs (e.g., miR-222-3p and miR-146a) and circular RNAs (e.g., circCCAR1 and circTRPS1) modulate the immune environment, contributing to immune evasion and tumor survival. PD-1: programmed cell death protein 1; uPAR: urokinase plasminogen activator receptor; ROS: reactive oxygen species; CTLA-4: cytotoxic T-lymphocyte-associated antigen 4. BioRender was used to create the figure.

In the intricate landscape of the TME, the presence of not only tumor cells but also resident stromal cells and infiltrating immune cells significantly influences tumor malignant properties and progression (39). Serving as messengers in intercellular communication, EVs emerge as crucial modulators shaping tumor growth, immunity, and drug resistance (40). This section delves into the functions of EVs derived from major cell types within the TME (**Figure 3**), elucidating their roles as regulators of tumors and inducers of immune responses.

### 3.1 Tumor cell-derived EVs

**Figure 4:**
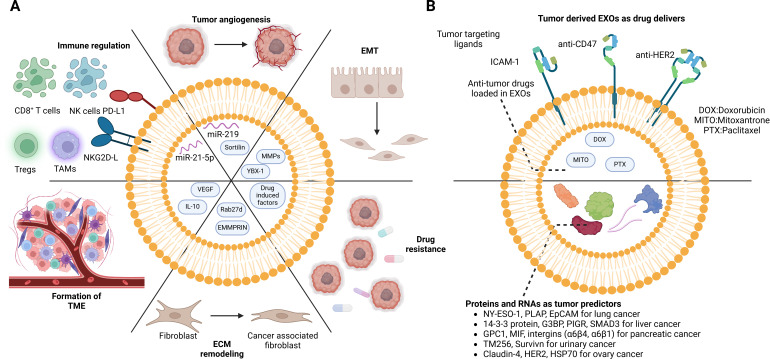
Dual role of cancer cell-derived EVs in the TME. Cancer cell-derived EVs not only interact with themselves but also shift the TME. **(A)** Exosomes from drug-resistance tumor cells induce anti-drug effects in drug-sensitive cells. The pro-growing factors in the exosomes directly stimulate tumor proliferation and metastasis. Moreover, the regulation of immune cells contributes to the immune escape of tumor cells. In addition, reprogramming of stromal cells facilitates tumor cell invasion. However, the EVs are also recognized as breakthroughs in tumor treatment. **(B)** Tumor-specific proteins and mRNAs can be detected in body fluid (plasma, serum, and urine), which can serve as cancer predictors. Taking advantage of the tumor-homing capacity of tumor-derived EVs, functional drugs can be loaded inside for precise delivery. DOX, doxorubicin; MITO, mitoxantrone; PTX, paclitaxel; NY-ESO-1, New York esophageal squamous cell carcinoma 1; PLAP, placental alkaline phosphatase; EpCAM, epithelial cell adhesion molecule; G3BP, GTPase activating protein (SH3 domain) binding protein; PIGR, polymeric immunoglobulin receptor; SMAD3, recombinant SMAD family member 3; GPC-1, glypican-1; MIF, macrophage migration inhibitory factor; HER-2, human epidermal growth factor receptor 2; HSP70, heat shock protein 70. BioRender was used to create the figure.

EVs originating from tumor cells play an essential role in influencing their own growth through autocrine mechanisms and shaping the behavior of adjacent cancer cells through intercellular communication (**Figure 4**). For instance, EVs derived from chronic myeloid leukemia cells contain TGFβ1, which promotes the growth of the producer cell through the activation of ERK, AKT, and anti-apoptotic pathways (41). Furthermore, impaired exosomal maturation and secretion due to the deficiency of vacuolar protein sorting protein 33b (VPS33B) significantly suppresses leukemogenesis (42). Tumor-derived EVs also act as promoting factors for adjacent cancer cells, exemplified by the transmission of oncogenic activity and increased proliferative capacity in glioma cells through the sharing of anti-epidermal growth factor receptor vII (EGFRvIII) via EVs (43). Additionally, these EVs are implicated in angiogenesis, a critical stage in tumor growth. They transport vascular endothelial growth factors (VEGFs) to endothelial cells, promoting the development of microvessels (44). CircRNA and mRNA in EVs also contribute to tumor progression in the TME (45–47). Exosomal circCMTM3 facilitates angiogenesis and tumorigenesis in hepatocellular carcinoma by regulating the miR-3619-5p/SOX9 pathway (45). let-7 g-5p derived from gastric cancer EVs drives M2 polarization in macrophages and contributes to the progression of gastric cancer (48). Moreover, the role of exosomal circRNAs in cancer chemotherapy resistance has been recognized (49). These circRNAs derived from drug-resistant cells are delivered to drug-sensitive cells and result in the resistance of one specific type of drug. For instance, the activation of the ciRS-122/miR-122/PKM2 axis promotes glycolysis and oxaliplatin resistance in colorectal cancer (50). The delivery of Circ-DNER induces the PTX resistance and cancer progression via the Circ-DNER/miR-139-5p/ITGB8 pathway in lung cancer (51).

### 3.2 Stromal cell-originated EVs

Stromal cells, including those aiding lymphocyte formation and maturation, play essential roles in shaping the TME. Exosomes derived from normal bone marrow-derived mesenchymal stem cells (BM-MSCs) inhibit the growth of multiple myeloma (MM) cells through increased levels of the tumor suppressor miR-15a (52). Similarly, the high content of exosomal miR-16 downregulated the expression of VEGF in breast cancer cells, which suppresses its growth (44). Conversely, stimulation of cancer cells tends to shift stromal cell-derived EVs toward pro-tumor phenotypes. For instance, fibroblasts stimulated by hepatoma cells exhibit a significant upregulation of SPOCK1/testican-1 pathways, promoting the progression of hepatoma cells (53).

Stromal cell-derived EVs contribute to tumor progression through various mechanisms, including cell proliferation, angiogenesis, and metastasis. They transfer certain RNAs and proteins to stimulate cancer cell proliferation. For example, BMSC-derived non-coding RNA triggered by DNA damage (NORAD) enhances osteosarcoma growth and invasion. Human umbilical cord mesenchymal stem cells (hucMSCs) transmit miR-100-5p, promoting malignancy development. These EVs also participate in tumor angiogenesis, promoting vascular density and tumor growth (54–56). EVs participate in tumor angiogenesis via transporting VEGFs and MMPs. It has been noted that hBMSC-produced EVs contain high levels of VEGF and CRCX4 mRNA, which encourage tumor angiogenesis and development *in vivo* (57). In addition, high levels of matrix metalloproteinase 1 (MMP1) in oral leukoplakia EVs (OLK-EVs) and oral squamous cell carcinoma EVs (OSCC-EVs) have been reported to be relevant to angiogenesis (58). Additionally, MSC-derived EVs facilitate tumor migration, influencing factors such as integrin expression and mesenchymal–epithelial transition (MET). For example, miR-374a-5p-loaded EVs of gastric cancer-derived MSCs target HAPLN1 to increase the expression of integrins in gastric tumors and promote gastric cancer cell migration (59). Given that EVs are natural nanocarriers with remarkable biocompatibility, their potential as drug delivery platforms has been extensively investigated by numerous researchers (60). Dormancy-inducing EVs from hBMSCs contribute to the acquisition of chemoresistance in metastatic breast cancer cells (61).

### 3.3 Pro-tumor immune cells

Immune cells play crucial roles in the TME, influencing tumor proliferation dynamics in various ways. During the initial stages, tumor cells recruit and activate immune cells, fostering an inflammatory environment that inhibits tumor growth. However, as the tumors advance, some immune cells may experience exhaustion or remodeling, leading to dysfunction and immunosuppression in the TME (62–64). The progress is regulated by the comprehensive intercellular communication network, in which EVs are largely involved by transferring significant signal molecules.

Though T cell-derived EVs are always recognized as antitumor NPs exhibiting a tumor suppression effect, it has been reported that EVs from exhausted CD8^+^ T cells assist tumor progression indirectly by impairing the proliferation of normal CD8^+^ T cells. Incubation of exhausted CD8^+^ T cell-derived EVs with normal CD8^+^ T cells results in reduced proliferation and activity, leading to an increased percentage of exhausted CD8^+^ T cells (65). Microarray technology reveals differential lncRNA expression between exhausted and non-exhausted CD8^+^ T-cell exosomes, highlighting the upregulation of tumor-promoting genes like SUMF2 and CHCHD1, and the downregulation of tumor-suppressing genes like UBXN10 in lncRNA of exhausted CD8^+^ T cell-derived EVs, indicating their potential in promoting tumor growth (65–69). Tumor-associated lymphatic endothelial cells (LECs) secrete EVs rich in miR-142-5p, which upregulates expression of 2,3-dioxygenase (IDO), leading to the exhaustion of CD8^+^ T cells via ARID2–DNMT1–IFN-γ signaling (70). In some instances, even activated CD8^+^ T cells may induce tumor metastasis via activation-induced cell death (AICD), which is mainly modulated by factor-related apoptosis ligand (FasL) (71, 72). FasL in EVs may indirectly participate in the process. It is reported that activated CD8^+^ T cells may secrete EVs containing high levels of FasL, increasing the quantity of cellular FADD-like IL-1β-converting enzyme (FLICE) inhibitory proteins, activating the ERK and NF-kB pathways, subsequently upregulating the expression of MMP9 in B16 murine melanoma cells (73). Natural killer (NK) cell exhaustion and B-cell exhaustion are primarily induced by consistent exposure to antigen stimulation, virus infection, and chronic inflammation, among others. During the exhaustion process, the inhibitor receptors like TIGIT, LAG-3, TIM-3, and PD-1 increase on the surface of the cells accompanied by the reduced expression of antitumor molecules like INF-γ, TNF-α, PFP, and granzyme. (74, 75) These findings confirm the reprogramming of exhausted NK cells and B cells, yet alteration of their EV content and function is rarely explored. These exhausted immune cell-derived EVs, however, may play a role in tumor immune escape, which deserves further research (76, 77).

Major function macrophages can be categorized into three phenotypes, namely, M1 macrophages, M2 macrophages, and tumor-associated macrophages (TAMs), of which M2 macrophages and TAMs promote tumor growth (78). Research on M2-EVs and TAM-EVs focuses on their pro-tumor capacity induced by regulatory molecules. For instance, miR-193b-3p in M2-EVs targets TRIM62, promoting progression and glutamine uptake in pancreatic cancer (79). By targeting GRK6, miR-3917 in M2-EVs promotes tumor progression in a lung cancer model (80). Similar effects driven by RNAs from M2-EVs or TAM-EVs have been observed in colon cancer, EOC, and prostate cancer (81–84). Exosomal RNAs adjust tumor function in alternative ways. Renal cell carcinoma aggression can be driven by miRNA-21-5p in M2-EVs via PTEN/Akt signaling (85). M2 macrophage polarization-associated lncRNA (lncMMPA) facilitates hepatocellular carcinoma malignancy by polarizing M2 macrophages and activating the glycolysis pathway (86). HIF-1α-stabilizing lncRNA from TAM-EVs can also regulate aerobic glycolysis in breast cancer cells (87). Drug resistance in pancreatic adenocarcinoma can also be induced by miR-365 in TAM-EVs. MiR-4443 derived from M2-EVs plays a role in the differentiation of naïve T cells into Treg cells in malignant pleural effusion, facilitating lung tumor growth (88). The same effect is also observed in EOC induced by miR-29a-3p and miR-21-5p in TAM-EVs (89). Other than RNAs, proteins like Arginase-1 from TAM-EVs also assist in cancer proliferation (88).

EVs derived from Tregs, Bregs, M2 macrophages/TAMs, and myeloid-derived suppressor cells (MDSCs) are not the primary regulators, but they also transfer significant messengers, mostly RNAs, such as microRNAs, lncRNAs, or circRNAs. As listed in **Table 2**, these EVs activate/silence different signaling axes, regulating key factors and then directly or indirectly boosting tumor growth or metastasis (90, 91). Treg-derived EVs contain several functional molecules that contribute to Treg suppressive activity. For example, exosomal CD73 converts adenosine-5-monophosphate to adenosine, activating the adenosine receptors on target cells’ surface, leading to immune modulation (92). The delivery of miR-146a-5p in Treg-derived EVs inhibit CD4^+^ T-cell growth (93). Moreover, miR-150-5p and miR-142-3p in Treg-derived EVs can modulate DCs’ cytokine constitution (94). It is reported that B-1a regulatory B cells (i27-Breg) can secrete EVs containing IL-27, suppressing and ameliorating uveitis (95). However, in the TME, IL-27 plays dual roles. It both supports CD4^+^ T cells’ proliferation and Th cells’ differentiation but promote tumor growth, invasion, and angiogenesis simultaneously (96). M2 macrophages and TAMs are all immune suppressive cells and their EVs serve a similar function. It is reported that M2 macrophage-derived EVs can foster tumor metastasis and increase vascular permeability in HCC via the delivery of miR-23a-3p, which targets phosphatase and tensin homolog (PTEN) and tight junction protein 1 (TJP1), promoting the secretion of GM-CSF, VEGF, G-CSF, MCP-1, and IL-4 from tumor cells, in turn facilitating M2 macrophage polarization (97). The cargoes found in MDSC-derived EVs have been shown to align with their role in mediating immune suppression by MDSCs (98). However, further in-depth research is necessary to assess the interactions between MDSC-derived EVs and other tumor-infiltrating immune cells, as well as their implications for cancer immunotherapy. Gaining a deeper understanding of the biological functions of MDSC-derived EVs will be crucial for their future therapeutic applications in cancer patients (99).

**Table 2:** EVs from immunosuppressive cells.

EV source	Cargoes	Signal pathway	Effect	Reference
Tregs	CD73	CD73-adenosine-AMP-adenosine receptors	Immune suppressive function	(92)
Tregs	IL-35	/	Coating bystander lymphocytes, causing non-Treg cell exhausting	(389)
M2 macrophages	miR-21-5p	miR-21-5p/YOD1/YAP/β-catenin	Facilitate CD8^+^ T-cell exhaustion	(390)
M2 macrophages	miR-21-5p	miR-21-5p-KLF3	Promote differentiation and activity of pancreatic cancer stem cells	(391)
M2 macrophages	miR-17-92	TGF-β1/BMP-7 pathways	Promoting hepatocellular carcinoma (HCC) proliferation	(392)
M2 macrophages	circRNA_CCDC66	circRNA_CCDC66-miR-342-3p-metadherin	Promoting the growth and mobility of colorectal cancer (CRC)	(393)
M2 macrophages	MISP	MISP/IQGAP1/PD-L1	Facilitated HCC cell immune escape	(394)
M2 macrophages	miR-143-3p	ZC3H12A/C/EBPβ axis	Promote CRC progression	(395)
Myeloid-derived suppressor cells	S100A9	S100A9/circMID1/miR-506-3p/MID1 axis	Facilitate castration-resistant prostate progression	(396)

### 3.4 Antitumor immune cells

As EVs mimic the functions of their donor cells, immune cell-derived EVs often exhibit antitumor potential with varying mechanisms. In this section, we categorize EVs based on their sources, including T cells, NK cells, DCs, and macrophages, clustering EVs with similar functions and shared mechanisms.

*T cells*

EVs produced by CD8^+^ T cells play a crucial role in modulating the communication between immune and tumor cells, thereby influencing tumor development. The interaction between PD-1 on the T-cell membrane and its ligand PD-L1 on the tumor membrane is a well-recognized communication pathway between T cells and tumor cells. Studies have shown that exosomal PD-1 produced by activated CD8^+^ T cells can reduce immunological dysfunction caused by PD-L1 in triple-negative breast cancer (TNBC) patients (100). Strategies involving CD8^+^ T cells treated with EVs containing specific cargoes have been explored to enhance their anti-PD-L1 capacity (101, 102). Additionally, the reduction of exosomal PD-L1 has been associated with an improved antitumor capacity of CD8^+^ T cells (103). By controlling the miR-765/proteolipid protein 2 (PLP2) axis, exosomal miR-765 produced by CD45RO-CD8^+^ T cells prevents the growth of uterine corpus endometrial cancer (UCEC) that is induced by estrogen (104). Similarly, CD4^+^ T cells regulate CD8^+^ T cells’ function via secretion of functional EVs. Exosomal miR-25-3p, miR-155-5p, miR-215-5p, and miR-375 from CD4^+^ T cells are responsible for CD8^+^ T cells’ activation. Compared with IL-2 as antitumor preparations in clinic, CD4^+^ T cell-derived EVs will not stimulate Tregs, which may suggest a promising new avenue for cancer immunotherapy by fostering a CD8^+^ T cell-mediated antitumor response (105). Meanwhile, CD4^+^ T-EVs are crucial for the activation, proliferation, and antibody generation of B cells, which is how humoral immunity is regulated (106). According to a recent study, CD4^+^ T cells can modify macrophages for enhanced cancer immunotherapy based on a stimulator of interferon genes (STING) signaling pathway (107).

#### 3.4.1 Natural killer cells

NK cells, being intrinsic tumor killers in the TME, have been explored in various immunotherapy strategies such as adoptive NK cell transfer, CAR-NK, and checkpoint blockade (108, 109). NK cell-derived EVs (NK-EVs) serve as powerful messengers, mimicking the antitumor function of NK cells. For instance, mRNA let-7b-5p in NK-EVs targets the cell cycle regulator CDK6, suppressing pancreatic cell proliferation (110). Cytolytic EVs enriched from primary NK cells possessed high apoptotic activity against HCT-116 colon cancer spheroids (111). NK-EVs also present a strong anti-hepatocellular carcinoma effect in subcutaneous and orthotopic animal models via inhibition of phosphorylation of serine/threonine protein kinases and activation of specific apoptosis markers (112). The potent ability of NK-EVs in anti-leukemia has also been verified (113). EVs from activated primary NK cells or NK-92 cells by IL-12, IL-15, and IL-18 are reported to have a better potential to penetrate and target solid tumors compared with those from inactive NK cell lines (114, 115). Furthermore, EVs from NK cells exposed to neuroblastoma cells augment the antitumor effect of EVs derived from cytokine-activated NK cells (116). The tumor-homing ability of NK-EVs makes them distinguished in drug delivery. NK-92 cell-derived EVs exhibit good targeting capacity in an NB tumor-bearing mouse model. Strong fluorescence is observed 6 h after injection, while EVs are observed in subcutaneous tumors in just 20 min after injection (117). However, NK-EVs can also be up-taken by normal cells and have shown cytotoxic effects in activated peripheral blood mononuclear cells (PBMCs) (118). Active NK cells with cytokines (e.g., IL-15) may promote its targeting ability (114, 119). In general, multiple investigations have concluded that there are no significant safety issues with NK-EVs in animal experiments (120–122).

#### 3.4.2 Dendritic cells

DCs are professional antigen-presenting cells (APCs) presenting antigens to T cells to stimulate their anticancer response. Its unique ability to induce primary and secondary immune response attracts investigation on EVs derived from DCs (DCs-EVs) for cancer treatment (123, 124). DCs-EVs are inextricably linked to the function of T cells. The presence of MHC-I, MHC-II, and costimulatory molecules such as CD86 in DCs-EVs stimulate T-cell immunity directly and indirectly (125). As a direct mechanism, DCs-EVs carry MHC molecules and costimulatory molecules and bind with the corresponding receptors including the TCR complex and coreceptor (CD4/CD8) to activate T cells via allorecognition *in vivo* (126, 127), yet some studies point out that this pathway does not occur in large quantities *in vitro* (126), and is less efficient than directly interacting with donor DC cells (128). However, increasing quantities of DCs-EVs may facilitate the direct-activation mechanism *in vitro*. An indirect mechanism activates T cells assisted by bystander APCs (129). EVs from mature DCs are transferred to naïve DCs, some are internalized, and the rest remain on the surface, which stimulates T cells (126, 130). Surface EVs can activate T cells by transferring MHC–peptide complexes, which are processed through the endosomal pathway. This process enables the transfer of antigen peptides from internalized EVs to the MHC molecules of recipient DCs (131), which is heavily influenced by integrins, ICAMs, and the activation status of the donor APC (132). DCs-EVs can also activate NK cells. It is reported that DCs-EVs can stimulate IFN-γ secretion by NK cells via exosomal TNF-α, Toll-like receptor (TLR)-4, and TLR1/2 (133, 134). Natural killer group 2-member D (NKG2D) ligands and IL-15Rα in DCs-EVs play a significant role in the direct activation of NK cells (135). The BAT3 molecule in EVs produced by DCs participates in the activation of NK cell-mediated cytokine release via binding to its ligand for the natural cytotoxicity triggering receptor 3 (NKp30) on NK cells (136).

#### 3.4.3 Macrophages

Similar to DCs, macrophages can serve as potent APCs. However, research on macrophage-derived EVs mainly focuses on their regularity function in the TME. M1 macrophage-derived EVs (M1-EVs) promote tumor apoptosis. According to research, canine M1-EVs can activate caspase-3 and caspase-7 to induce tumor death. Additionally, the expression level of CCR4, Foxp3, and CTLA-4 is reduced in canine peripheral mononuclear cells cocultured with tumor cells (137). A recent study highlights the role of human cytosolic glycyl-tRNA synthetase (GARS1) on the M1-EVs membrane in tumor apoptosis via interacting cadherin6 (CDH6) on the cancer cell surface. Additionally, the extracellular cadherin subdomains 1–4 of the cadherin EGF LAG seven-pass G-type receptor 2 (CELSR2) interact specifically with the N-terminal WHEP domain-containing peptide region of GARS1 to cause M1 divisiveness of macrophages and activate the RAF-MEK-ERK pathway for M1-type cytokine production and phagocytosis (138). Cytokine signaling 3 in alveolar macrophage-derived EVs inhibits STAT3 activation, suppressing the progress of lung cancer (139).

## 4 The potential application of EVs in cancer treatment

With the enhanced understanding of EVs’ role in the TME, the application of EVs as a tumor immunotherapy agent has been realized. The potential of EVs as a tumor vaccine has been explored since EVs possess immunogenicity. As a cell product, EVs inherit the characteristics of donor cells, which may serve as alternative ACTs since EVs have a lower side effect than cells. EVs can activate and regulate the immune system with outstanding targeting capacity and editable flexibility, attracting researchers to transform them into antitumor drugs and drug delivery platforms.

### 4.1 EVs as a cancer vaccine

**Figure 5:**
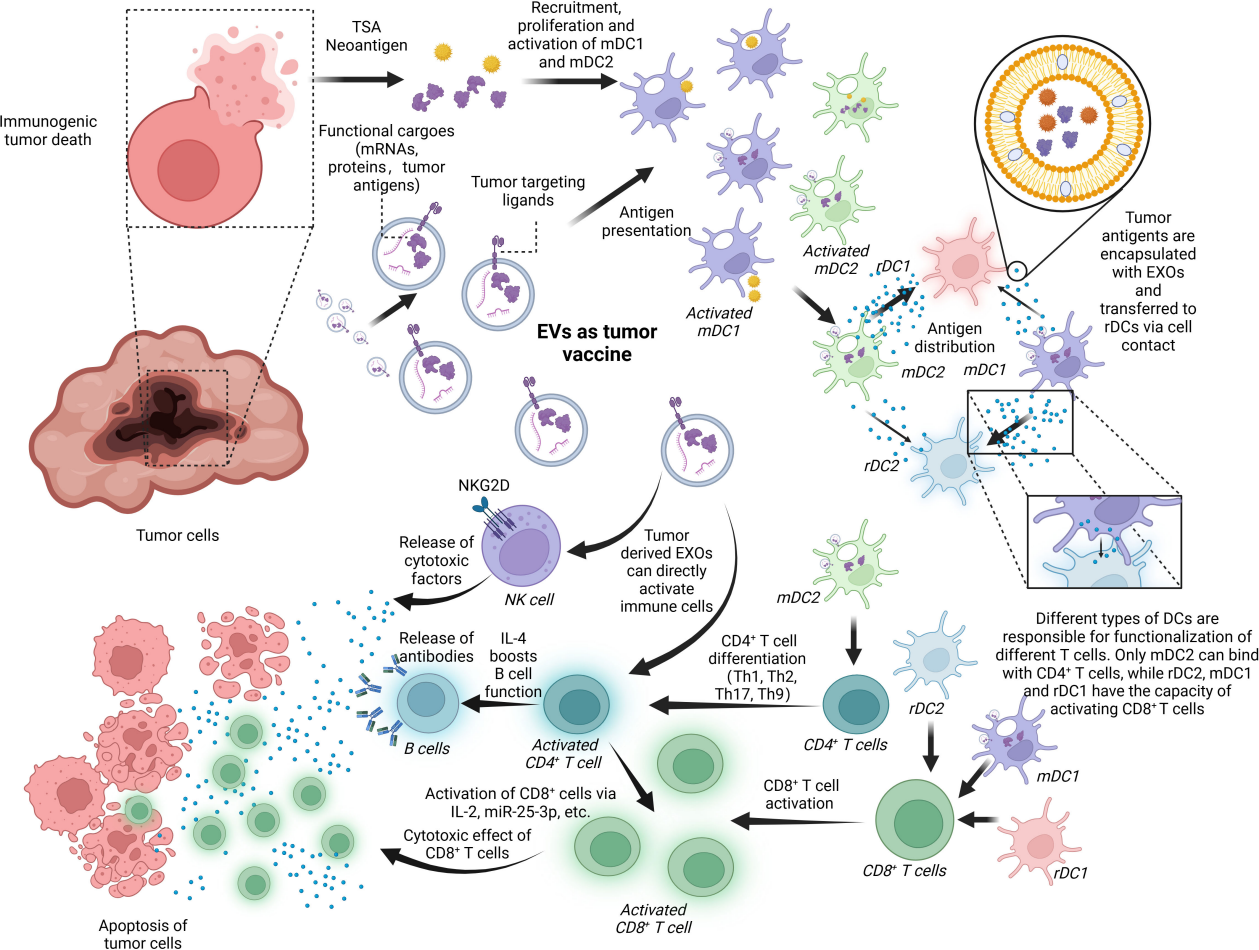
EVs as a tumor vaccine. EVs are deeply involved in the progress of tumor immunoregulation and, thus, have the potential to serve as a tumor vaccine for the next generation. Tumor-derived EXOs or the debris of dead cancer cells involves tumor-specific cargoes like tumor neoantigens and some tumor markers like HSP 70, EGFR, and K-Ras. These factors can activate mDCs including mDC1 and mDC2, which are responsible for transferring the antigens to rDC1 and rDC2. It is noted that the antigen-distributing process is based on surface contact and transferring of EXOs containing tumor antigens. rDC1 tend to receive more EXOs than rDC2 from mDC2, and mDC1-derived EXOs have a preference for rDC2. In the last, rDC1, rDC2, and mDC1 can work together to promote CD8^+^ T-cell activation while just mDC2 can boost CD4^+^ T-cell function. In addition, tumor-derived EXOs are capable of activating immune cells like NK cells, CD8^+^ T cells, and CD4^+^ T cells directly without assistance of APCs. BioRender was used to create the figure.

Cancer vaccines activate the immune system against tumor cells. In the process, a high volume of high-quality tumor antigens are presented to DCs and activate them to promote CD8^+^ T cells and CD4^+^ T cells, in which EVs are mainly involved (140). EVs, mirroring the functions of their donor cells, can present exosomal tumor antigens to DCs, eliciting tumor-specific CD8^+^ T cells and CD4^+^ T cells (141–143). The inclusion of tumor neoantigens within EVs further contributes to the development of tumor vaccines (11). DCs-EVs as antigen presenters have also found application in immunotherapy, serving as agents of vaccines. Additionally, these EVs can activate immune cells through the regulation of cytokines (144). **Figure 5** shows the basic mechanism of an EV-based cancer vaccine.

Tumor-derived EVs provide antigen to APCs. MHC class I and class II are found on the surface of tumor-derived EVs, which is responsible for antigen presentation (145). In lung cancer, EGFR, K-Ras, basigin, carcinoembryonic antigen-related cell adhesion molecule 6, claudin1, claudin3, and RAB family proteins are found to be differentially expressed (146). EVs derived from colorectal cancer can interact with DCs, significantly enhancing immune responses by lowering the antigen presentation threshold for activation at the mucosal level (147, 148). APC-derived EVs inherently rely on MHC compatibility, necessitating a precise match with the MHC haplotype. Conversely, EVs derived from tumor cells transcend this limitation, as they do not require MHC haplotype matching. This pivotal characteristic enables the development of anticancer vaccines that are cell-free and can be universally applied, eliminating the need for individualized engineering for each patient. Furthermore, these EVs harbor tumor antigens that transcend the confines of a single cancer type, hinting at their potential to confer protection against a diverse spectrum of cancers (149). It is noted that exosomal heat shock protein 70 (HSP70) can active DCs and monocytes to trigger immune response and stimulate NK cells to release granzyme B, inducing tumor apoptosis (150, 151). In conclusion, HSP70 can potentially act as an antigen on the surface of EVs to trigger antitumor responses. In certain stages of tumor progression or under proper stimulation, tumor-derived EVs can activate immune cells. For instance, it is reported that bladder cancer cell-derived EVs can boost CD8^+^ T-cell function via cytokine regulation (144). Tumor antigens on these EVs also efficiently activate immune responses (152). EVs derived from tumor cell exposure to methotrexate (MTX) impair the antitumor effect of neutrophils via internalization and degradation of PD-1 in the lysosomes (56). Similarly, antitumor drugs induced highly enclosed HSPs in EVs from human hepatocellular carcinoma cells, which induce inhibitory receptor CD94 and reduce activating receptors CD69, NKG2D, and NKp44, efficiently enhancing NK cell cytotoxicity and granzyme B production (153). However, their application relies on advanced isolation techniques capable of precisely extracting tumor-derived EVs from complex body fluid.

DC-derived EVs serve as the main source of exosomal antigen provider (154). As the most potent APC *in vivo*, DCs are responsible for antigen uptake and T-cell activation (155). Migration DCs (mDCs) in the TME encapsulate the tumor antigen via EVs and transfer them to resident DCs (rDCs) in draining lymph nodes, which is responsible for activating CD8^+^ and CD4^+^ T cells (156). rDC1, rDC2, and mDC1 are responsible for CD8^+^ T cells’ activation while only mDC2 can make CD4^+^ T cells work. The delivery of antigen also relies on the intercellular transferring of EVs. T cells cannot be activated if the EVs are unable to form (157). Nowadays, DC-derived EVs have been welcomed by many researchers as tumor vaccine carriers. Tumor neoantigen can be loaded in the nanovaccine delivery platform built via DC-derived EVs for individualized immunotherapies. The nanovaccine has demonstrated efficient cargo loading and sustained cargo delivery to the lymph nodes, leading to robust antigen-specific T-cell- and B-cell-mediated immune responses with excellent biosafety and biocompatibility. Notably, the delivery of the neoantigen-EV nanovaccine significantly inhibits tumor growth, extends survival time, delays tumor recurrence with long-term immunological memory, and eradicates lung metastasis in therapeutic, prophylactic, and metastatic B16F10 melanoma models, as well as in therapeutic MC-38 models. Furthermore, the EV-based nanovaccine exhibits a synergistic antitumor response that outperforms liposomal formulations, owing to the presence of EV proteins. Collectively, the research presents enhanced strategies for cell-free vaccines and highlights the potential of EV-based nanoplatforms in cancer immunotherapy and personalized nanotechnology. These findings pave the way for the rapid generation of individualized nanovaccines for clinical use (158). Immunogenic cell death (ICD) inducers are encapsulated within DC-derived EVs and used as a tumor vaccine against breast cancer cells. This approach demonstrates potent antitumor activity in both a mouse model and human breast cancer organoids by enhancing the activation of cDC1s *in situ*, thereby boosting subsequent tumor-reactive CD8^+^ T-cell responses (159).

Non-antigenic immune adjuvants are essential in cancer immunotherapy, as they enhance immunogenicity and promote antigen presentation, thereby improving the immune response against weakly immunogenic tumors. Previous research has shown that adjuvant-loaded EVs are more effective than administering free adjuvants. Leveraging these insights, as well as the unique properties of EVs, they have been investigated as nanocarriers for the targeted delivery of adjuvants (160, 161). Immune cell-derived EVs can serve as adjuvants themselves, influencing the proliferation and differentiation of immune cells (162). During infection, the amount of circulating phosphatidylserine^+^ (PS) EVs increases, actively modulating CD8^+^ T-cell responses and preferentially interacting with activated, but not naive, CD8^+^ T cells (163). The researchers either take advantage of the inherent homing ability of EVs or modify their surface for precise targeting (164, 165). Additionally, EVs’ capability to deliver adjuvants directly into the cytosol through membrane fusion exhibits significant potential for enhanced immune activation (166). Currently, CpG DNA (167), lipid adjuvants (168, 169), cytokine adjuvants (170–172), HSPs (153, 173), and Gram-negative bacterial outer membrane vesicles (OMVs) (174) are applied as adjuvants prioritizing activation of CD4^+^/CD8^+^ T cells. However, safety concerns persist because adjuvants can trigger inflammatory reactions, which may include fever, ulcers, or even potentially life-threatening cytokine storms.

### 4.2 Engineered EVs in ACTs

ACT is a type of cancer treatment that genetically modifies T cells to detect and destroy cancer cells. This approach enhances or changes the intrinsic immune function of T cells, increasing their effectiveness in combating cancer (175). T cell receptor-engineered T (TCR-T) cell therapy and chimeric antigen receptor T (CAR-T) cell therapy are two major trends in ACT, involving genetically engineering T cells to express receptors that specifically target tumor antigens (176, 177). TCR-T cells possess receptors binding TSAs both on the tumor surface and inside tumor cells while CAR-T cells target cancer cells via membranal proteins and kill them without undergoing the antigen-presenting process (178). Traditionally, the engineered T cells are amplified *in vitro* and infused into the patient’s body, yet the effect is limited because of the reduced penetration ability and potential side effects like cytokine release syndrome (CRS), immune effector cell-associated neurotoxicity syndrome (ICANS), and secondary cancers, among others (179–181). Altering CAR-T cells with CAR-T cell-derived EVs may be a solution boosting its antitumor effect. First, CAR-T-derived EVs are stable particles with a limited lifespan and are unable to proliferate, which may reduce the side effects induced by CAR-T cells, especially CRS. Second, CAR-T-derived EVs are non-cell preparations with low antigenicity, making its application in third-party settings as an off-the-shelf product favorable. Third, CAR-T cell-derived EVs can penetrate the tumor barrier, which may be a solution towards solid tumors (182). CAR-T cell-derived EVs show great potential as direct agents in immunotherapy. These EVs equipped with EGFR and HER2-specific CARs demonstrate a strong capacity against EGFR+ and HER2+ tumor cells in xenograft models. Compared to CAR-T cells, CAR-T cell-derived EVs lack the expression of PD-1, making their antitumor activity resistant to suppression by recombinant PD-L1 treatment (183).

CAR-NK-EVs have also caught researchers’ attention in recent years. Compared with CAR-T cells, CAR-NK cells are less likely to induce life-threatening CRS. Furthermore, CAR-NK therapy is anticipated to be more cost-effective, as NK cells can be sourced from PBMCs, NK cell lines, and human pluripotent stem cells (hPSCs) (184). Moreover, NK EVs will not harm normal cells (118). It has been reported that NK EVs can induce the death of target cells through two major mechanisms: ligand–receptor interactions and plasma membrane fusion (185, 186). Recently, CAR-NK-EVs have been engineered to enhance antitumor therapy by targeting and disrupting the iron death defense mechanism. By modifying the transferrin receptor-binding peptide and expressing CAR on the surface of the EVs, these engineered vesicles can effectively cross the BBB and release therapeutic molecules precisely at the intended sites and times (187). However, the interaction between NK-EVs, other immune cells, and tumor cells is comprehensive and the mechanism has not been clearly delineated; thus, the application of CAR-NK-EVs is worth further exploration.

### 4.3 EVs as an en route drug delivery platform

Given that EVs are natural nanocarriers with a remarkable biocompatibility, their potential as drug delivery platforms has been extensively investigated by numerous researchers.

EVs are recognized as safe vesicles. Native EVs *in vivo* are reported to undergo reduced hepatic clearance (36, 188). This helps reduce the administration dosage and decrease the potential side effects (34). EVs exhibit exceptional biocompatibility and reduced immunogenicity. In one study, EVs extracted from bovine milk show limited liver and kidney toxicity and no significant increase in histamine concentration (189). Furthermore, early-phase clinical trials have reported mild to moderate side effects of EV-based NP delivery platforms, supporting their safety in clinical applications (15, 190).

Their great penetration power brings EVs to almost everywhere *in vivo*. There are several barriers in the human body that protect some important organs or tissues from foreign bodies and maintain their normal function. Yet, the barriers prevent most of the drugs from entering these locations (191). The BBB is an intricate and highly selective barrier in the human body. It serves to safeguard the brain and maintain the stability of the central nervous system (CNS). This barrier is primarily composed of endothelial cells that form a tightly joined monolayer, covering the brain’s capillaries. What is worse, brain tumor cells tend to format the BTB (192). EVs can cross the barrier via several mechanisms like receptor-mediated transcytosis, lipid raft-mediated endocytosis, and micropinocytosis (21). Given this character, EVs are considered promising carriers targeting tumors in the brain. Ginseng-derived exosome-like nanoparticles (GENs), composed of phospholipids and various bioactive components, are currently being evaluated for their ability to stimulate antitumor immune responses in T cells and Tregs, with the aim of inhibiting tumor progression. Their enhanced targeting ability to the BBB and glioma shows a significant therapeutic effect, demonstrating strong efficacy in recruiting M1 macrophage expression within the TME. GENs are proved to be successful candidates for glioma therapeutics in both *in vitro* and *in vivo* studies, indicating excellent potential for inhibiting glioma progression and regulating TAMs (193). Fruit-derived EV-engineered structural droplet drugs (ESDDs) are created by programming the self-assembly of fruit-derived EVs at the interface of DOX@squalene-PBS, significantly enhancing their antitumor efficacy against glioblastoma. The blood–testis barrier is among the most restrictive blood–tissue barriers found in mammals. It separates the seminiferous epithelium into two distinct areas: the basal compartment and the apical (or adluminal) compartment (194). EVs have been applied in delivering drugs to testis. Sertoli cell-derived small extracellular vesicles (SC-sEVs) can cross the BTB and enter germ cells. By loading miR-24-3p inhibitors into these vesicles, the nano-drug SC-sEV@miR-24-3p inhibitor is created, which efficiently delivers the miR-24-3p inhibitor to germ cells. In a mouse model of gossypol-induced asthenozoospermia, treatment with the SC-sEV@miR-24-3p inhibitor significantly enhanced sperm motility, increased the success rate of *in vitro* fertilization, and improved blastocyst formation rates. As expected, it also increased the litter size in asthenozoospermia mice. These findings suggest that the SC-sEV@miR-24-3p inhibitor could be a promising clinical treatment for asthenospermia (195).

The potent targeting capacity of EVs boosts precision medicine. EVs can be passively accumulated in the TME via the enhanced permeability and retention (EPR) effect. The EPR effect refers to the phenomenon where NPs of appropriate sizes preferentially accumulate in tumor tissues compared to normal tissues, leading to an extended retention time of the NPs within the tumor area (196, 197). This phenomenon occurs because the abnormal blood vessels found in tumors enhance vascular permeability (198). NPs ranging from 20 to 200 nm in size can infiltrate the interstitial space due to the misaligned and defective endothelial cells (199). Additionally, the clearance of NPs from the TME is often delayed due to the limited lymphatic drainage present in these areas (200). Moreover, EVs can be actively targeted to tumor cells by the ligand–receptor connection. On the surface of tumor cells are tumor markers such as carcinoembryonic antigen (CEA) for colorectal cancer (201), carbohydrate antigen 125 for ovarian cancer (CA125) (202), and neuron-specific enolase (NSE) for neuroendocrine tumors (NET). Furthermore, taking advantage of the prosperity in the field of membrane-editing technology, the EVs’ membranes are engineered to enhance their targeting ability for precise cytotoxic effect and limited harm to normal cells. Engineered DCs-EVs, bound with membrane anchor lysosome-associated membrane glycoprotein 2b (Lamp2b) and brain-specific rabies viral glycoprotein (RVG), showcase the potential of EVs as targeted drug delivery systems. After tail vein injection of the EVs, knockdown of BACE1 mRNA and protein is demonstrated in the brains of mice (19). EVs can also be reprogrammed to promote their accumulation in the TME. It is reported that low pH, a significant feature in the TME, reprograms tumor EVs for enhanced homology via a glycolipid self-aggregation-based mechanism, which sheds light on the exploitation of environment-responding EVs (203). The engineered EVs derived from M1 macrophages are conjugated with dibenzocyclooctyne-modified antibodies targeting CD47 and SIRPα (aCD47 and aSIRPα) via a pH-sensitive linker. These EVs are designed to accumulate in the acidic TME and specifically target tumor cells by recognizing the interaction between aCD47 and CD47 on the tumor cell surface (204). Additionally, the magnetic field gradient is an alternate noninvasive technique to improve targeting efficacy. EVs derived from macrophages are loaded with drugs and iron oxide NPs, which spatially regulate the absorption of EVs and drugs by cancer cells *in vitro* (205). The self-targeting capacity of tumor-derived EVs can also be employed for precise targeting (206). EVs derived from tumor-repopulating cells (TRCs) isolated from three-dimensional fibrin gels enhance the efficiency of drug delivery. Compared to EVs derived from tumor cells cultured on conventional tissue-culture plastic, TRC-derived EVs, when intravenously injected into mice with tumor xenografts, show increased accumulation in tumor tissues, improved crossing of blood vessels, and deeper penetration into the tumor parenchyma. They are also preferentially taken up by highly tumorigenic TRCs. Additionally, the cytoskeleton-related protein cytospin-A plays a crucial role in regulating the softness of TRC-derived EVs. Modulating the mechanical properties of these EVs could improve the delivery efficiency of anticancer drugs (207). The EV preparation can be delivered to tumor sites via drainage tubes for some metastatic tumors like malignant pleural effusion (MPE) or malignant ascites, or injected directly into the superficial solid tumors like melanoma. For most tumors, intravenous injection is the most common administration method. Since tumor capillary permeability (~780 nm) is larger than that in normal tissue (5–8 nm), it is hard for tumor-derived EVs (100–1,000 nm) to reach normal tissues while they can enter tumor parenchyma easily (208–210). However, the potential oncogenesis risk of tumor cell-derived EVs limits their application as theriacal molecules and drug delivery, yet an attempt to apply tumor cell-derived EVs to deliver drugs is on trial (listed in **Table 3**).

**Table 3:** EVs as a nanoparticle delivery platform.

Cell source	Size	Isolation method	Engineer strategy	Functional molecules	Function	Year of publication	Reference
Human lung carcinoma A549 and hepatocarcinoma H22 cell	~210 nm	Ultracentrifugation	Incubation	DOX, methotrexate, cisplatin, PTX	Transfer pro-tumor M2 macrophages to antitumor M1 phenotype Release IFN-β Reverse drug resistance of soft tumor-repopulating cells	2016, 2023	(397, 398)
Human breast cancer cell MDA-MB-231	~100 nm	Ultracentrifugation	Incubation	Bovine milk lactoferrin	Inhibit MDA-MB-231 cancer cell growth	2023	(399)
Epithelial cancer cell MCF-7	~140 nm		Electroporation (eliminate endogenous cargoes) Sonication, hypertonic loading, electroporation (load cargoes), and incubation	Gemcitabine (GEM), miR-21 inhibitor	Potent targeting ability Higher antitumor efficiency	2023	(400)
Murine breast cancer 4T1	–	Differential ultracentrifugation	Electroporation	Let-7i, miR-142 and, miR-155	Inhibit tumor growth Promote IFN-γ and granzyme B production ability of cytotoxic T cells	2021	(401)
Myeloid leukemia cell line K562	~58 nm	Total exosome isolation reagent, Invitrogen™, No. 4478359	Genetic modification	IL-15, IL-18, and 4-1BBL (TNFSF9)	Increase cytotoxicity of NK cells Promote NK cell proliferation	2017	(402)
Melanoma cell B16	–	Ultracentrifugation	Transfection	Early secretory antigenic target-6 (ESAT-6)	Suppress tumor growth	2016	(403)
Murine B cell (M12.4)	<150 nm	Ultracentrifugation, anti-CD63 immunomagnetic capturing and Exoquick-TC™	HiPerFect and FuGENE^®^ HD (transfection)	MiR-155 inhibitor	Reduction in LPS-induced TNFα production	2014	(404)
B cell	<180 nm	Differential ultracentrifugation	Epstein–Barr virus (transduction)	Glycoprotein gp350	Inhibit EBV infection in B cell	2011	(405)
Human umbilical cord blood-derived mononuclear cells (hUCB-MNCs)	~131 nm	Differential ultracentrifugation	Exo-Fect Exosome Transfection Reagent (transfection)	MiR-124-3p	Protect dopaminergic neurons in the substantia nigra and striatal fibers	2022	(406)
Platelet	120–150 nm	Chromatography	Extrusion, freeze/thaw, or sonication	DOX	Kill breast cancer cell	2023	(407)
Platelet	100–300 nm	Size exclusion chromatography	Incubation	PTX	Kill breast cancer cell	2023	(408)
Platelet	~140 nm	Differential ultracentrifugation	Electroporation	Yap1 protein	Regenerate tendon	2023	(409)

Moreover, surface modification of EVs through cellular machinery techniques facilitates tumor targeting and intercellular transformation (34). For example, modified NK-EVs can be obtained by exogenous and endogenous alterations. Exogenous modifications involve loading drugs like sorafenib or cisplatin onto NK-EVs, which enhance apoptosis in TNBC and reactivate NK cell functions against drug-resistant ovarian cancer, respectively (211, 212). Engineered NK-EVs with small interfering RNA (siRNA) and hydrophobic photosensitizer Ce6 present cytotoxic effects towards tumor cells via reactive oxygen species (ROS) and conscripted immune cells (122). Endogenous modifications achieved enriched specific cargo through lentiviral transduction into the parent cells. Lentiviral transduced NK92MI cells enrich BCL-2 siRNAs in EVs, enhancing their intrinsic apoptosis in breast cancer cells (121). Though NK cells share similar functions with T cells, rare attention has been paid to CAR-NK-derived EVs, which may be endogenous modification methods of the next generation.

M1-EVs, when engineered, demonstrate enhanced abilities to inhibit tumor growth and regulate the immunosuppressive TME. Engineered M1-EVs deliver RSL3 as a ferroptosis inducer, which disrupts redox equilibrium to increase the oxidative stress-triggered robust ferroptosis of tumor cells (213). Docetaxel-loaded M1-EXOs polarize naïve M0 macrophages toward the M1 phenotype as opposed to the M2 phenotype by using mitochondrial function (214). M1-EVs can also load therapeutic agents, like catalases, DNA damage repair inhibitors, and anti-PD-L1, which simultaneously target tumor hypoxia, cancer DNA damage, and T-cell function (215). In addition, EVs derived from macrophages are potent drug delivery systems. Engineered M1-EVs have been created by functionalizing the membrane with chemical excitation source CPPO and photosensitizer Ce6, as well as encapsulating the hydrophobic prodrug AQ4N. These modified M1-EVs penetrate the BBB, induce M2-to-M1 polarization, and increase hydrogen peroxide (H_2_O_2_) levels. The reaction between H_2_O_2_ and CPPO activates Ce6, generating large amounts of oxygen species to achieve chemiexcited photodynamic therapy (CDT). AQ4N also converts into toxic AQ4 in the hypoxic TME, inducing apoptosis of glioblastoma multiforme (GBM) (216). AS1411 aptamer-modified macrophage exosomes are also utilized to coat the sonosensitizer indocyanine green, enhancing the sonodynamic therapy of glioblastoma (217). Furthermore, the modified macrophages’ EVs demonstrate strong antitumor activity, indicating the significant potential of macrophages as sources of EVs (218).

The excellent feature of inflammatory chemotaxis makes neutrophil-derived EVs (NE-EVs) a remarkable drug for targeting tumors. NE-EVs are loaded with DOX for targeted glioma therapy, which penetrates the BBB and reacts to inflammation. Additionally, NEs-EVs/DOX intravenous infusion effectively slows tumor growth and lengthens survival in a mouse model of glioma (219). In another study, NE-EVs are decorated with superparamagnetic iron oxide nanoparticles (SPIONs) to improve the tumor-targeting capacity, enhancing the antitumor effect of DOX. These EVs induce tumor apoptosis without affecting normal cells, exhibiting superiority in targeting and efficacy compared to normal NE-EVs (220).

## 5 Challenges and opportunities for clinical application

The progress in the field of EV studies attracts more and more researchers to investigate the potential of EVs in clinical settings. However, laboratory conditions differs from those in clinic, and the focus varies. In the laboratory, researchers mostly care about the characteristics and functions of EVs, yet the cost of large-scale synthesis, the safety of EV preparation, and storage methods, among others, are of great importance in clinical settings. Moreover, standards and guidelines illustrating EV application remain to be established. The current plights and potential solutions are reviewed in the following section.

### 5.1 Large-scale synthesis of EVs to be amenable in the clinic

As mentioned above, strategies applying EVs as therapeutic agents have been explored in-depth. However, the widespread clinical application requires large-scale synthesis of EVs. Notably, scaling out and scaling up can optimize the cell culture process and thus contribute to the vast production of EVs.

The scale-out of the culture system refers to the cultivation of more cells in a limited space. For instance, the Integra CELLine Culture System increases EV yield to 10.06 ± 0.97 mg/mL compared with 0.78 ± 0.14 mg/mL by traditional culture (221). This technology is designed for scalable production of EVs from adherent cell lines, like cancerous bladder cells (222). Regretfully, this platform is not suitable for stromal cells since it maintains cells at high densities for prolonged periods of time. Another strategy for vast production is the automatic release of EVs in hollow fiber bioreactors. In this system, cells are grown on the surface of semi-permeable fibers and release EVs to flowing supplemented media. However, previous research reported the occurrence of cell differentiation and increased cell density after 6 weeks of culture. This phenomenon leads to the low EV yield of a single cell (223, 224). Optimizing collection frequency may solve such challenges. In particular, a combination of hollow fiber bioreactor and size exclusion chromatography/tangential flow filtration enables the production and enrichment or purification of clinical-grade EVs at a moderately vast scale (225). In addition, the application of hyperflasks reduces manual operations during cell culture and media harvest and promotes the production of EVs, yet compared with 2D flasks, they are not economical (222). The CellBIND^®^ surface is pretreated with oxygen-containing functional groups and carries a net negative surface charge. Bioreactors are commonly utilized for large-scale production due to their dynamic monitoring systems, which are advantageous for GMP processes (226). Xeno-free cell culture significantly improves EV production by reducing cell doubling time, increasing EV yield, and achieving up to 97% removal of contaminating proteins. Specifically, a 10% pooled human platelet lysate (HPL)-based, EV-depleted medium effectively supports the production of human MSC-derived exosomes while maintaining their characteristic surface markers, morphology, viability, and *in vitro* differentiation potential (226, 227).

Meanwhile, the scale-up strategy increases EV yield by cultivating cells on microcarriers in stirred tank bioreactors. This 3D culture method outperforms the traditional 2D culture method in the aspect of silencing siRNA loading (228). Additionally, physical stimulation, including hypoxia (229), low pH (162, 230), heat shock (231), or ultrasound (232), can improve the production of EVs. Serum deprivation is another stimulation to boost EV yield that prevents the contamination of serum-derived EVs and particles (233, 234). Of note, although EVs produced by stimulation are often reported to display similar physical characteristics with those produced without stimulation, their protein and RNA contents may be different, which could undermine the process of EV preparations (235).

In addition to optimizing the cellular culture, ideal cellular sources can also facilitate the large-scale production of EVs. One potential source could be embryonic stem cells (ESCs). ESC exhibits the capability of almost unlimited self-renewal and offers sufficient EV sources for clinical application (236). According to previous research, EVs secreted by ESCs display satisfactory antitumor properties. Human red blood cells (RBCs) are another ideal cellular source. Specifically, group O-RBCs could be utilized as universal donors for the vast production of EVs. This is because group O-RBCs are devoid of DNA and are available in blood banks (237).

### 5.2 Clinic oriented purification strategies to be optimized in the future

Purification strategies have always been a major problem affecting the process of the clinical application of EVs (238). The selection of purification strategies has a direct impact on clinical effects. On the one hand, it is significant to eliminate contaminations in EV preparation including the unexpected EVs, liposomes, proteins, and RNAs. For instance, some EVs are extracted from culture medium of cancer cells *in vitro* for their well-known tumor-targeting capacity, yet the pro-tumor EV subtypes may harness the therapeutic effect. What is worse, it is noted that tumor-derived EVs play a critical role in modulating the TME, promoting tumor growth, metastasis, immune evasion, and even drug resistance via various means (239, 240). On the other hand, some so-called “contaminations” ought to be kept in EV preparation. One reason is that it is impossible to isolate the single-component EVs or one designated subtype of EVs from complex EV groups of body fluid or culture medium (241). One major obstacle in the field of EV isolation is the large scale of co-isolated lipoproteins sharing similar characteristics such as density, size, and component (242, 243). It is noted that the application of density-gradient ultracentrifugation for EV purification results in the co-isolation of LDL and HDL due to the similar density, and a SEC-based isolation strategy will lead to contamination of chylomicrons (244). Even the purification strategy combining the SEC purification step and the differential ultracentrifugation cannot avoid the presence of LDL in plasma EV preparations since the LDL particles have a higher concentration by several orders of magnitude than EVs in human plasma (245). Ultracentrifugation, the main applied isolation method, is also impaired by LDL and HDL residues due to the sedimentation rate difference of EVs and HDL/LDL (246). Though there is a new technique applying the styrene-maleic acid (SMA) copolymer to selectively break down lipoproteins, it has not been widely applied and its adverse effect on SMA remains unknown (247). Another reason is that the so-called “contamination” may facilitate efficacy in some cases. In a study, researchers discovered that the presence or absence of EVs did not impact the ability of human mesenchymal stem cell (hMSC)-conditioned medium to promote angiogenesis and wound healing *in vitro* while much smaller soluble factors like VEGF play a more important role in the progress. However, when applied in far higher concentrations than those presented in conditioned medium, the hMSC-derived EVs also exhibit wound-healing capacity (248). The research can be seen as a warning, urging scientists to reconsider the complex relation between EV and non-EV factors and the potential experimental hazards when conducting experiments regarding EV bioactivity. In a word, it is both unlikely and unnecessary for researchers to achieve “complete purification”, namely, isolating the single-ingredient EV during preparation. For the clinical application of EVs, we can focus on isolating EV groups including specific subtypes of EV and non-EV factors. The EV groups should satisfy clinical needs, avoid potential adverse effects, and entail reduced purification cost.

Different isolation methods lead to different outcomes. Despite starting with the same source, different processing procedures may lead to the various mixtures of co-isolates and EV subsets. In the study, higher pERK/ERK ratios are observed after stimulation of SEC-EVs than after stimulation of UC-EVs, which indicates the significance of optimizing isolation strategies in the clinic (249). Another study reports that different isolation methods leave different contaminations in HEK293T-derived EV preparation. These media component contaminations result in the suspicious error that HEK293T-derived EVs possess anti-inflammatory bioactivity (250). These studies reveal that different isolation methods applied in different research studies influence the validity and accuracy of the result and even may lead to false outcomes.

Standard purification strategy matters in clinical settings. The impact of isolation methods has been mentioned above. The heterogeneity of isolation methods hinders the quantification and comparison of the results (251). It is noted that 80% of studies simply conduct ultracentrifugation to isolate the EVs (242, 243). Ultracentrifugation has been recognized as the gold standard for EV isolation and is efficient in enriching EV fractions, allowing for the collection of additional larger EV components (252). However, it has restricted the processing volume due to the limited thin loading zone (253). Furthermore, the requirement of expensive equipment and well-trained technicians restricts its wide application. In addition, prolonged exposure to ultracentrifugal forces can negatively impact the structure and biological function of isolated EVs, making them less suitable for downstream applications like EV-based functional studies and drug development (254). According to MISV2018, you cannot obtain both high yield and high purity in EV production (18, 255). Thus, we ought to establish a standard purification strategy, which is a series of isolation technique combinations catering different EV sources (culture medium or body fluid). Moreover, such a strategy entails guidelines that will evaluate existing and new methods and purify EVs in order to confirm their usage in different scenarios. The strategy aims at stipulating standard, economic, and efficient purification methods for mass production and clinic use.

### 5.3 Allowing stabilized long-term storage during EV preparation

Another noteworthy problem preventing EV preparations from widespread clinic application is storage. Although numerous studies have examined the properties of, and the roles played by, EVs *in vivo* and their potential in immunoregulation, drug delivery, and biomonitoring, they keep the EVs or raw materials containing EVs (e.g., culture medium, body fluid, and extracts) for a short period, ignoring the changes in the active ingredient in EVs during long-term storage (256, 257). According to the research, even if the EVs are carefully enriched, isolated, and purified, improper preservation strategies may still result in EV fusion or crack, protein aggregation, or degradation, leading to failure in EV preparation (256, 258). Like other biopharmaceuticals, the application of EVs will involve a series of procedures including transfer, storage, and disposal, requiring the exploration of economic storage strategy and the definition of quality period (256, 258, 259).

In light of this lack of standard procedures for EV storage and regarding research, current studies have not reached a consensus (260). The application of cryoprotective agents (CPAs) like trehalose (261, 262) or dimethyl sulfoxide (263, 264) is suggested by some authors. Researchers have discovered that adding 25 mM trehalose to the isolation and storage buffer for pancreatic beta-cell exosome-like vesicles narrows the particle size distribution and increases the number of individual particles per microgram of protein. In macrophage immune assays, beta-cell EVs stored in trehalose consistently show higher TNF-alpha cytokine secretion stimulation indexes, indicating better preservation of biological activity (261). Furthermore, polyacrylamide gel electrophoresis (PAGE) analysis demonstrated that both proteins and RNA within EVs are preserved after lyophilization when trehalose is present. Lyophilization has minimal impact on the pharmacokinetics of Gaussia luciferase (gLuc)-labeled EVs following intravenous injection into mice (262). Cryopreservation of platelets with DMSO leads to the release of platelet microvesicles (PMVs) and a significant increase in thrombin generation and procoagulant activity (TG-PCA) compared to liquid-stored platelets (LSPs) (263). The morphology of EVs cryopreserved using DMSO is similar to SEM images of fresh EVs. Although the sizes and shapes of a certain percentage of EVs are preserved, DMSO is unable to maintain the morphology of all vesicles in the sample (264). Another widely applied method for long-term preservation is cryopreservation including freezing and lyophilization (262, 265). The EVs are usually recommended to be preserved at −80℃ for long-term preservation and at 4℃ for temporary storage (266). A series of studies discuss the effect of different temperatures or the speed of freezing or thawing. Despite encouraging findings, some results remain conflicting, and comprehensive studies that compare different storage strategies simultaneously are still lacking. Additionally, most studies have analyzed samples after relatively short time periods (e.g., hours, days, or occasionally weeks). Those that have examined samples after longer preservation periods have primarily focused on the storage of biofluids rather than isolated EVs (265, 267–272). Lyophilization makes its progress in vacuum to protect the easily oxidized components. Moreover, lyophilization reduces the water in EV samples, which enhances their stability and reduces the risk of contamination, facilitating transportation and extending storage duration (273). Lyophilization without a cryoprotectant results in the aggregation of exosomes derived from B16BL6 melanoma cells, while adding trehalose, a cryoprotectant, prevents this aggregation. PAGE analysis reveals that trehalose protects the proteins and RNA of the exosomes during lyophilization. The procedure has little effect on the pharmacokinetics of Gaussia luciferase (gLuc)-labeled exosomes after intravenous injection into mice. Additionally, lyophilized exosomes retain the activity of loaded gLuc and immunostimulatory CpG DNA for approximately 4 weeks, even when stored at 25°C (262). The spray-dry technique is another technique preserving EVs. When an EV solution is atomized in a drying chamber, the moisture quickly evaporates once in contact with hot air and leaves dry powder. During this process, atomization pressure and outlet temperature are factors that influence the stability of EVs. Compared to lyophilization, spray drying is a continuous process that can achieve one-step formation, making it more economical and suitable for large-scale production (274). However, like lipid nanoparticles (LNPs), shear stress, liquid interface expansion, and stress caused by thermal dehydration during the collection process may harness the EV membrane (275, 276). In recent years, some researchers try to preserve EVs in gelatin methacryloyl hydrogel (GelMA) (277). Since the irregular Brownian transport of EVs is the cause of membrane fusion and the inactivation of its contents (278–281), GelMA can encapsulate EVs, limiting their random movement and reducing their aggregation, thus improving their stability. The good biocompatibility, the ability to be administered without affecting EV activity, and the well-established preparation techniques that enable synthesis or commercial availability highlight its potential for clinical application (277).

In conclusion, there are six key points in EV storage. (1) Freeze–thaw reduction. It is reported that the freeze–thaw cycle decreases EV yield and increases particle size due to membrane fusion and protein loss. EV transportation should follow the principle of minimizing freeze–thaw cycles. If the EVs being transported are stored under frozen conditions, it is recommended to use sufficient dry ice for transportation. When transporting freshly isolated EVs, it is advised to use adequate ice packs and deliver them to the destination as quickly as possible (282, 283). (2) Application of screw caps and rubber seals to reduce the impact of freeze-drying during the storage of EVs. The rubber seals enhance the tightness of screw caps, separating EVs from contaminants from the air and reducing oxidation (284, 285). (3) Utilizing low-adsorption materials to store EVs. It reduces loss of EVs and assists in keeping the key molecules on the EVs’ surface (286). (4) To prevent ice crystal formation and reduce low-temperature precipitation, seal the container with sealing film, aliquot the EV samples, and quickly freeze them in liquid nitrogen. Store at −80°C or below, and thaw at 37°C (287). (5) Purified EVs can be temporarily stored at 4°C, but should not be kept for more than 48 h. Furthermore, it is recommended to prioritize storing unextracted samples at −80°C because untreated samples are better stored at −80°C compared to purified EVs (282, 283, 288). (6) Storage conditions and duration have a significant impact on EVs. A higher storage temperature and a longer storage time contribute to fewer EVs remaining in the sample (260).

### 5.4 Potential of EVs as biomarkers in clinical practice

Recently, applying EVs as a biomarker and as a diagnostic and prognostic predictor has been another promising clinical application (289). There are several reasons for EVs to be an excellent disease indicator. First, EVs are intercellular vesicles transmitting bioactive molecules regulating cell development, differentiation, and function, which can serve as sensitive and specific biomarkers (290). Second, secreted by most cells *in vivo*, EVs exhibit a high concentration in most bodily fluids, which means they can be easily captured and tested, especially large EVs (291). Third, accumulating studies have revealed the relation between changed EV content and certain diseases (292). For example, it is reported that the combination of EV TDP-43 levels and EV 3R/4R tau ratios can assist in diagnosing frontotemporal dementia (FTD), FTD spectrum disorders, and amyotrophic lateral sclerosis (ALS). EV tau ratios are low in progressive supranuclear palsy (PSP) and high in behavioral variant frontotemporal dementia (bvFTD) with tau pathology. EVs TDP-43 levels are elevated in ALS and in bvFTD with TDP-43 pathology. Both markers effectively discriminate between diagnostic groups, achieving area under the curve values greater than 0.9. They also differentiate between TDP-43 and tau pathology in bvFTD. Additionally, both markers strongly correlate with neurodegeneration, as well as with clinical and neuropsychological indicators of disease severity (293). Through selective reaction monitoring/multi-reaction monitoring (SRM/MRM), EphA2 on urinary EVs presents significant expression differences between patients with bladder cancer/non-malignant hematuria and healthy controls. The subsequent research also shows that EVs-EphA2, which promotes the proliferation, invasion, and migration of bladder cancer cells, exhibits strong diagnostic performance, with a sensitivity of 61.1% and a specificity of 97.2% (294). In addition, EVs may have some prognostic value. It is reported that tRNA-derived small RNAs (tsRNAs) are specifically enriched in salivary EVs of ESCC patients with high sensitivity (90.50%) and specificity (94.20%). According to the bi-signature Risk Score for Prognosis (RSP), patients with a high RSP have significantly shorter overall survival (OS) (HR 4.95, 95% CI 2.90–8.46) and progression-free survival (PFS) (HR 3.69, 95% CI 2.24–6.10) compared to those with a low RSP. Moreover, adjuvant therapy is found to improve OS (HR 0.47, 95% CI 0.29–0.77) and PFS (HR 0.36, 95% CI 0.21–0.62) only in patients with a high RSP, but not in those with a low RSP (295). Another study indicates that EV-derived B7-H3 and B7-H4 emerge as noninvasive predictors of survival in patients with metastatic NSCLC treated with ICIs, functioning independently from their expression in tumor tissue. Interestingly, an increase in PD-L1^+^ EVs is primarily linked to disease progression, while a rise in B7-H3^+^ EVs seems to be associated with a positive response to ICI treatment (296). Moreover, EVs can guide drug application. For example, it is reported that small extracellular vesicles (sEVs) carry multiple inhibitory immune checkpoint proteins, creating a potentially targetable adaptive mechanism that suppresses antitumor immunity. It sheds light on the role of sEVs in tumor drug resistance mechanisms, which is helpful in improving the patient response rate of immune checkpoint blockade therapy (297). Another comprehensive review reveals the role of EVs in assisting drug resistance and the possible methods that target EVs to overcome tumor drug resistance (298). However, the application of EVs as biomarkers is still in its infancy and there are some practical problems that remain to be solved.

The source and isolation techniques of EVs determine their application. Currently, EVs as noninvasive biomarkers are usually extracted from blood and urine (299); CSF and saliva are also included in some studies (300, 301). Blood is the most abundant EV source among bodily fluids, yet its complex components and viscosity bring up significant challenges for EV isolation (233); thus, kits are not recommend for blood–EV isolation since co-precipitated proteins and liposomes may contaminate the EVs (302–304). Size exclusion chromatography (SEC) and ultracentrifugation are effective methods for separating protein from EV components with a higher yield and purity compared to other methods, yet there are still residual lipoprotein particles. Furthermore, EVs separated by ultracentrifugation tend to have lower yields, which may not meet the minimal testing dose (305). It is recommended that a combination of different methods can be applied to remove liposomes and proteins when isolating blood EVs (306). However, an improper isolation method may damage the target protein, leading to a false result (307). Urine, as another widely applied bodily fluid for disease detection, is considered the most suitable biological specimen for research on urinary system-related tumors due to its direct connection with the urinary system (308). Unlike blood, urine samples can be collected in larger volumes with a lower protein content. Given the characteristics of urine samples, ultrafiltration is commonly used to concentrate the urine, removing soluble contaminating proteins and obtaining concentrated EV-containing solution. Subsequently, methods such as SEC or precipitation are employed to isolate relatively pure EVs (309). CSF is recognized as the best bodily fluid reflecting brain and spinal cord conditions, and CSF EVs are helpful in maintaining healthy nervous system function (310). CSF can be collected via brain puncture and lumbar puncture. Brain puncture can collect a large volume of samples but may be contaminated by blood. Lumbar puncture collects 20 to 25 mL of sample at a time; it is usually free of blood and can ensure a replicable strategy (311). It is important to exclude blood from CSF samples. Thus, centrifugation of the lumbar CSF sample before freezing and storage is recommended to eliminate any potential cellular contamination that could interfere with EV research. The sampling position, method, and the subsequent processing strategy should be identical when making effective comparison in medical research or clinical settings (312). As to CSF EV isolation, filtration and SEC are recommend techniques since they work well on small-volume samples (313). Saliva has its advantages of being easily collected, noninvasive, and safe (314). Saliva EVs not only reflect the health condition of the adjacent tissue of salivary gland (295, 315), but also can be a biomarker for other tumor diseases like breast cancer, pancreatic cancer, and lung cancer (316–319). However, saliva is primarily secreted by the sublingual, submandibular, and parotid glands, and the composition of saliva varies depending on the sampling location and time (320). Thus, when collecting saliva EVs, it is necessary to either standardize the sampling location or collect saliva from all locations (320). Moreover, the composition of saliva also changes at different times of the day; thus, sample timing is also important (321). Notably, eating, drinking, smoking, and vigorous exercise are prohibited within 1 h before sampling to ensure that saliva EVs are not contaminated (322).

It is noted that race may have an impact on EV content. This should be taken into consideration when determining the threshold. For example, exosomal miR-1304-3p has been identified as the most upregulated mRNA in African American breast cancer patients, showing a significant difference when compared to Caucasian American patients (323). However, limited attention is paid to the field, partly because of the difficulty in sample acquisition and insignificance in content difference.

### 5.5 Turning the tables: using EVs as weapons

Attempts to apply EVs in preclinical and clinical settings have emerged recently with satisfactory results (324). The application mainly focuses on utilizing EVs as diagnosis and prognosis biomarkers and modifying EVs as therapeutic agents (325–327).

Compared with healthy people, cancer patients tend to present high levels of EV production (328). Their correlation with tumor not only helps with early diagnosis but also indicates potential targets for tumor immunotherapy (329). It is noted that the early detection of tumors is crucial for effective cancer immunotherapy (330). For instance, miRNA-200-5p, miRNA-378a, miRNA-139-5p, and miRNA-379 are proven to be noninvasive sensitive biomarkers in the diagnosis and screening of lung cancer (331). It is also found that postoperative blood samples from pancreatic ductal adenocarcinoma (PDAC) patients showed reduced levels of exosomal Sox2ot expression, which plays a role in tumor development and may serve as a valuable prognostic marker for pancreatic cancer (332). Understanding how EVs package their cargo, release it into bodily fluids, and maintain stable levels in health and disease is crucial for developing powerful biomarkers to monitor disease onset and progression (324, 333).

Besides biomarkers, EVs can also be therapeutic agents. EVs derived from DCs originate from either immature or mature DCs activated by cytokines, such as recombinant interferon-γ. The injection dose typically ranges from 8.5 × 10¹¹ to 4.0 × 10¹³ EVs containing MHC class II molecules. To stimulate an immune response in cancer patients, these DC-derived EVs, loaded with tumor antigens, are administered subcutaneously. Clinical trials using these EVs have been conducted for melanoma and non-small cell lung cancer, showing similar safety outcomes. However, in the case of non-small cell lung cancer, MAGE-specific T-cell responses have been observed (334). These tumor antigens, such as carcinoembryonic antigen, can be obtained directly from cancer patients by harvesting ascites-derived EVs. A phase I trial demonstrated that this method is safe and well-tolerated. Moreover, in the group receiving ascites-derived EVs combined with granulocyte–macrophage colony-stimulating factor, a tumor-specific cytotoxic T lymphocyte response is observed (335). Manipulating EV content and production also aids immunotherapy. In a study, researchers discovered that EVs extracted from malignant ascites contained the MET oncogene, which enhanced tumor invasiveness. Furthermore, inhibiting the secretion of these EVs suppressed tumor progression (336).

Co-delivery of chemotherapy drugs or siRNAs also boosts tumor immunotherapy. EVs have been extensively utilized in the treatment of various malignancies (337). It is reported that repeated injection will not harm to body (328). Moreover, their immune compatibility and biocompatibility make them well-suited for therapeutic applications (338). Two clinical trials (NCT01854866 and NCT02657460) are investigating the application of chemotherapy drugs to treat patients with malignant pleural effusion. In the preclinical study and the NCT01854866 trial, MTX and cisplatin are used as anticancer drugs, respectively. Results from the preclinical study show a higher survival rate in MTX application compared with cisplatin (339). KrasG12D siRNA has been explored as a potential anticancer therapy for patients with metastatic pancreatic cancer. In another study, mesenchymal stromal cell-derived EVs have been proposed as a therapeutic approach, as outlined in clinical trial NCT03608631. In an orthotopic pancreatic tumor model, targeting tumor-initiating cells (TICs) with MSC membrane-derived nanovesicles, known as “nano-ghosts,” loaded with a CXCR3 antagonist improved treatment efficacy and delayed tumor recurrence when combined with gemcitabine. Since MSC-derived nano-ghosts may preferentially home to tumors and specifically target the TIC population, we propose utilizing them as “Trojan horses”. A promising approach for overcoming treatment resistance, particularly in desmoplastic cancers such as pancreatic adenocarcinomas, is nano-ghost-based therapy (340).

Insults to donor cells can produce EVs with distinct features. Generally speaking, tumor cell-derived EVs inhibit tumor immunotherapy and promote tumor growth and metastasis. However, there are some exceptions (341, 342). UV-treated tumor cells produce EVs devoid of HSPs but enriched with abundant genomic and mitochondrial DNA fragments. These EVs can serve as vaccines stimulating DCs via cGAS/STING signaling. This finding suggests a new tumor cell-free vaccine strategy with promising potential for clinical applications (343). Radiation-treated cell-released EVs show the capacity to suppress murine brain metastasis via the reprograming of the TME by inhibiting the MAPK pathway (342).

To date, the heterogeneity of EVs may have an impact on their clinical application. The heterogeneity of EVs likely reflects their size, composition, functional effects on recipient cells, and cellular source. Size heterogeneity may be induced by the uneven invagination of the limiting membrane during formation and flaws in isolation techniques (344, 345). Even after purification, EVs are observed to exhibit variable abundance of cargoes, including mRNAs and proteins (244, 346). Subsets of EVs may function differently in one set of the isolated EVs: one set may promote cell survival while another induces cell apoptosis (347). The heterogeneity of EVs may influence the results of EV studies, yielding false-positive or false-negative results. Furthermore, advanced techniques in EV purification and classification ought to be developed to distinguish the subtypes of EVs with different functions.

## 6 Conclusion and perspective

As a fast-growing and exciting new field developing rapidly in the last 20 years, EVs have attracted the attention of numerous researchers and its application has flourished. EVs not only play a significant role in immunoregulation but also have become a promising tool in diagnosis, prognosis, and target therapy. In this review, we have summarized current EV isolation strategies, classified standards, and researched their pros and cons in a clinical setting. Although the biogenesis of EVs has been fully explained, EV isolation and modification techniques have been developed, and some researchers have attempted to take advantage of EVs to build drug delivery platforms or EV-based immunotherapy strategies, there is still a long way to go as regards their clinical application. However, our understanding of EVs is inadequate, which hinders us from fully exploiting the potential of EVs. Most studies regarding EV biogenesis are conducted *in vitro*, yet the relevance of mechanisms *in vivo* remains to be investigated. Some studies suggest targeting the formation and release of specific types of EVs. However, it is still unknown whether all cells share the same mechanism in secreting EVs and influencing the target cells, which may hinder the development of the targeting therapy.

The interaction of EVs *in vivo* has been studied extensively by numerous researchers. Current research reveals that EVs are deeply involved in multiple cell activities and intercellular communication. Presenting pro-tumor or immunosuppression factors, tumor or regulatory immune cell-derived EVs participate in the process of TME shaping and tumorigenesis, tumor proliferation, tumor metastasis, and tumor drug resistance. Antitumor immune cell-derived EVs also present a strong capacity in promoting tumor apoptosis or activating other immune cells. However, some details in the EV regulatory process remains unknown, which may induce the “butterfly effect” due to the complexity of the immune system, ultimately leading to an incorrect result. Despite widespread exploration, our understanding of EVs’ roles in the TME remains superficial; continued research is needed to understand the content, function, and responsiveness of EVs. The heterogeneity of EVs in the TME suggests potential applications in monitoring tumor growth, especially considering the influence of treatment, tumor classification, grading, and stage. Moreover, the EV environment decides whether EVs are either “good” or “bad”, indicating that researchers should pay attention to not only the physicochemical properties and content of EVs but also the biogenesis, position, and activity of a specific type of EV *in vivo*. Utilizing naïve EVs as antitumor drugs is a promising strategy, leveraging their reduced tumorigenic risk and heightened biological activities. In a word, a more comprehensive and systematic view of how EVs work in the human body is required in future research.

The potential of EVs as a drug delivery platform has received widespread attention. A limited number of clinical trials have attempted to add EVs in their drug delivery systems (348). The EV source, loaded cargoes, targeted disease, and drug administration have witnessed a diversification. These trials demonstrate the safety of EVs in a clinical setting. However, with the development of techniques, these trails are not up-to-date enough to meet current need. Production transformation is encouraged to bridge the lab–clinic divide. In the future, certain cell strains may be selected as engineered EV providers for industrial production. According to recent research, MSCs and cytotoxic immune cells (e.g., CD8^+^ T cells, NK cells, and M1 macrophages) are promising candidates, yet their indications remain to be further explored. Another strategy caters the trend of personalized medicine, emphasizing sampling the EV source from the patient. For example, when treating cancer patients, researchers may build a customized EV-based drug delivery platform via tumor cells from the patients themselves. The tumor-derived EVs should be processed to eliminate tumorigenicity yet keep their self-targeting capacity. However, the strategy may not be economic and efficient enough to be applied immediately, thus relying on technique optimization in the future. Furthermore, its safety and efficacy remain to be verified.

Advancements in EV-relevant techniques and guidelines are also required for further application. EV isolation and modification techniques have been developed and some researchers have attempted to take advantage of EVs to build drug delivery platforms or EV-based immunotherapy strategies, but there is still a long way to go with regard to their clinical application. It is necessary to exploit different isolation strategies aimed at various application scenarios. When it comes to the large-scale production of EV preparations, it is important to develop isolation strategies, while balancing cost and purity and ensuring effectivity and safety. When researching EVs or applying them as biomarkers, combining low-volume isolation strategies that are less harmful to target molecules on EVs may be the solution (349). The modification of EVs to serve as an effective drug-targeting nanoplatform establishes the groundwork for the development of “next-generation” anticancer nanomedicines. The development of international guidelines, quality classifications, and good manufacturing practices (GMP) rules is essential to ensure the safety and efficacy of EV therapy in clinical settings. The establishment of standards also helps in the comparison of different research outcomes.

The production of GMP-grade EVs depends on several factors, including the type of applied cells, the culture environment, the cultivation system, the dissociation enzyme, and the culture medium. After production, further purification is typically required, which is generally carried out in a three-step process. A third critical aspect of GMP-compliant EV production is the development of reliable identification methods, encompassing both their physical structure and bioactivity characteristics. Five types of cells have been applied in GMP EV production. However, the lack of guideline enrolling new types of cells applicable in GMP EV production may inhibit further exploration and clinical application. Culturing systems, purification systems, and characterization have been previously mentioned, yet a more comprehensive and approved guideline should be determined. The common flaws of the existing techniques in producing and purifying EVs include a limited scale, the contamination of heterogeneous EVs/proteins/lipids/nucleic acids, and the high cost. The application of engineering cell lines, flasks/bioreactors with a modified surface, and an enlarged surface area may boost large-scale synthesis. To avoid contamination, culture medium without EVs component ought to be used. Proper stimuli will further promote EVs yield. The lack of regulation may result in poor preparation quality and confusion in drug application. Guidelines and consensus documents could be developed by experts in the field annually to address the issue.

To fully realize the potential of EVs as biomarkers for diagnosis and prognosis, progress in assorted hardware and software is necessary. Currently, most attempts using EVs as biomarkers remain in the laboratory, and the testing procedures are too complex to be applied in the clinic. Microfluidic chips with highly sensitive detectors may be the new trend in the field of EV biomarkers. To calculate the reference value, credible algorithm and software should also be developed, and a larger-scale clinical trial is significant for data collection. In conclusion, a standard procedure for data collection, analysis, and reporting should be established for EV-specific regulatory approval.
